# Astragalin attenuates caerulein-induced acute pancreatitis by targeting the NLRP3 signaling pathway and gut microbiota

**DOI:** 10.1186/s40643-025-00977-3

**Published:** 2025-12-03

**Authors:** Yan Jia, Yuxin Shi, Jie Wang, Honghui Liu, Hanyue Wang, Yilin Huang, Ya Liu, Peiyan Chen, Jie Peng

**Affiliations:** 1 Department of Gastroenterology, Xiangya Hospital, Central South University, Changsha, 410008 China; 2 National Clinical Research Center for Geriatric Disorders, Xiangya Hospital, Central South University, Changsha, 410008 China

**Keywords:** Astragalin, Acute pancreatitis, Gut microbiota, Network pharmacology, NLRP3 signaling pathway

## Abstract

**Background:**

Acute pancreatitis (AP) has caused great concern worldwide due to its serious threat to human health. Astragalin is a bioactive natural flavonoid compound with several pharmacological activities, but it remains unclear about its effect on AP. The objective of this experiment was to explore the mitigating efficacy of astragalin on caerulein-induced AP model and examine the underlying mechanisms.

**Methods:**

Following the assessment of astragalin’s direct effects on pancreatic acinar cells using an in vitro AP model, an in vivo mouse model was established to further validate its efficacy and elucidate the underlying mechanisms. Pancreatic histopathology, amylase, and lipase levels of mice were observed to determine the optimal therapeutic dose of astragalin. The network pharmacology and RNA sequencing technology were used to reveal the possible targets and pathways. Subsequent molecular docking and western blot were conducted to validate the association between astragalin and key target molecules, as well as the NLRP3 signaling pathway. Combined with metagenomics and metabolomics analysis, the astragalin effective gut microbiota-metabolite-gene network was constructed. Moreover, fecal microbiota transplantation experiments were performed to clarify the importance of gut microbiota in astragalin-mediated alleviation of AP.

**Results:**

The results showed that astragalin attenuated caerulein-induced injury in AR42J cells in vitro. Consistent with these findings, in vivo experiments revealed that astragalin treatment significantly improved pancreatic pathological injury, cell apoptosis, and systemic inflammatory response in AP mice, particularly at high doses. The integrated analysis of network pharmacology and transcriptomics revealed that the NLRP3 signaling pathway was a key molecular pathway, which was further validated using western blot. Docking analysis showed that 12 target genes had good docking activity with astragalin. More intriguingly, it was found that astragalin could reverse gut microbiota dysbiosis by restoring microbial diversity, altering bacterial community composition, and modulating key metabolic pathways. Specifically, astragalin-effective correlation networks were constructed with *Lachnoclostridium* sp. YL32, *Roseburia intestinalis*, *Ruminococcus gnavus*, *Lachnospiraceae bacterium* Choco86, *Anaerobutyricum hallii*, etc. as the core strains, 22 metabolites, including 5-Methoxytryptophan, D-Serine, L-Tryptophan, L-Methionine, etc. as core metabolites, and NLRP3 pathway-related genes as the main regulatory targets. Furthermore, fecal microbiota transplantation experiments confirmed the involvement of gut microbiota in AP remission.

**Conclusion:**

Collectively, these findings identify astragalin as a promising therapeutic agent for AP, targeting both the NLRP3 signaling cascade and gut microbial homeostasis.

**Graphical Abstract:**

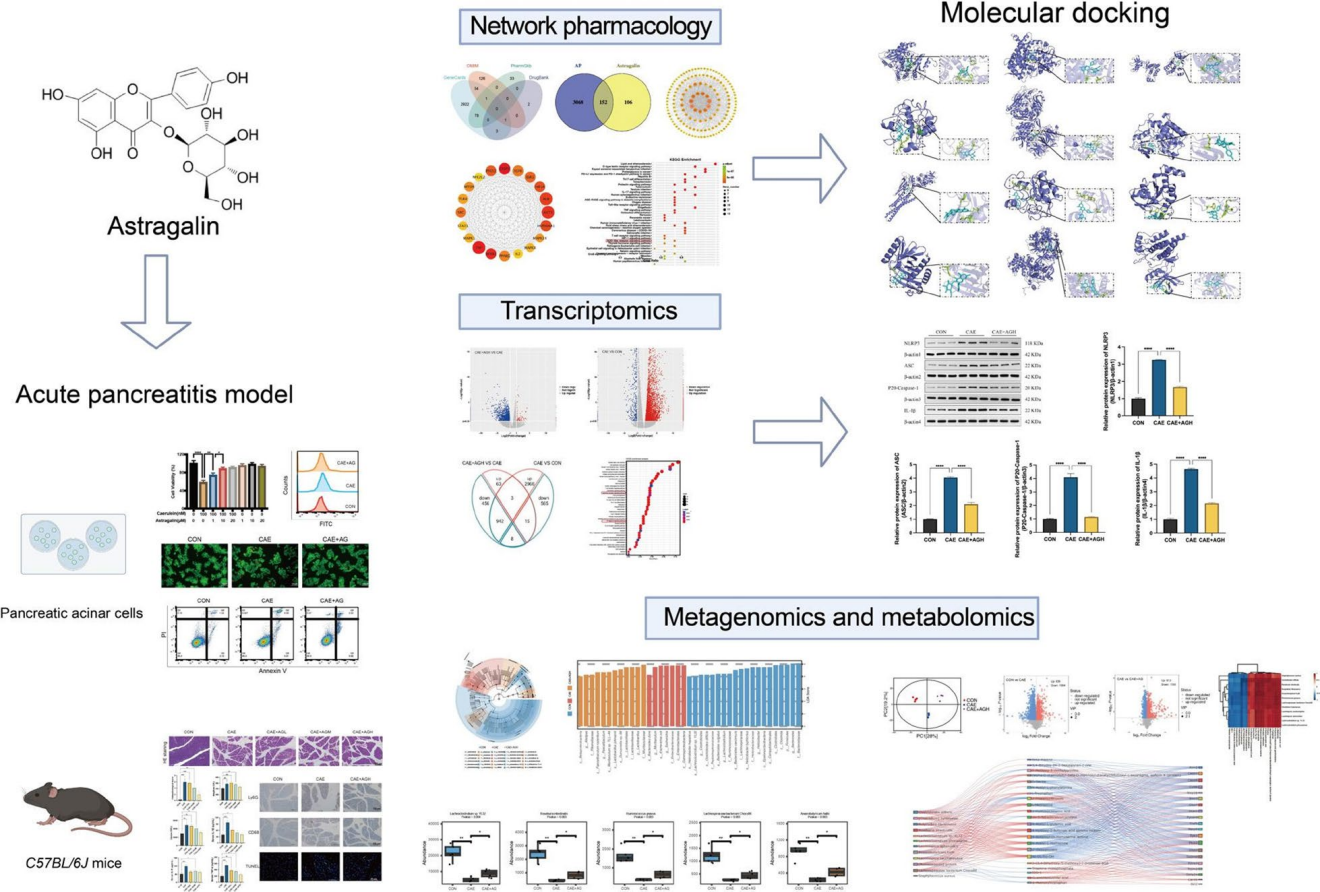

**Supplementary Information:**

The online version contains supplementary material available at 10.1186/s40643-025-00977-3.

## Introduction

Acute pancreatitis (AP) is a common gastrointestinal disease that frequently demands emergency hospitalization. It is characterized by a marked elevation in pancreatic enzyme levels and acute abdominal pain (Frossard et al. 2008). The pathogenesis of AP remains complicated and obscure, among which premature activation of trypsinogen, pathological calcium signaling, mitochondrial dysfunction, endoplasmic reticulum stress, and abnormal autophagy have been extensively studied and are known to have a significant impact on the onset and progression of AP (Biczo et al. 2018; Criddle et al. 2007; Gukovskaya et al. 2017; Lugea et al. 2011). A series of clinical trials have been conducted targeting key molecules in the pathogenesis, however, there are no specific drugs for AP treatment. Therefore, it is necessary and urgent to explore novel therapies that are more promising and effective.

Owing to its unique theoretical system and effective therapeutic methods, the prevention and treatment of illnesses have long been addressed by traditional Chinese medicine (TCM). Nowadays, numerous studies on the application of TCM targeting AP and other inflammatory diseases have highlighted its superiority, which is mainly attributed to the regulation of the integrity and function of the organism without limitation to local organs (Wang et al. 2011; Xia & Deng 2013). Astragalin (AG) is a natural flavonoid derived from numerous plants and is well known for its medical value. To be specific, its pharmacological effects include neurotrophic, cardioprotective, anti-inflammatory, antioxidant, antidiabetic, and anticancer. Prior research has indicated that AG alleviates inflammatory diseases by regulating NF-κB, Protease-Activated Receptor 2 (PAR2), inducible Nitric Oxide Synthase (iNOS), and mitogen-activated protein kinase (MAPK) (Peng et al. 2020; Soromou et al. 2012; Zhang et al. 2017). Besides, AG possesses antidepressant action by modulating NLRP3 inflammasome deactivation (Tong et al. 2020). However, whether AG has a protective effect against AP and its specific mechanism remains unclear.

Technologies like network pharmacology, transcriptomics, metabolomics, and metagenomics are currently employed to study the multi-target effects of drugs on disease models. Network pharmacology takes a comprehensive view of biological systems, examining the interactions between drugs and diseases (Yuan et al. 2017). This approach, which uncovers molecular relationships between drugs and therapeutic targets, is consistent with the holistic principles of TCM. Transcriptomics, a technology that analyzes the full set of RNA transcripts, provides insight into gene structure and function, revealing how drugs affect disease mechanisms. It is commonly used to detect differentially expressed genes (DEGs) in individuals (Yang et al. 2020). Therefore, the combined application of network pharmacology and transcriptomics enhances understanding of herbal components and their molecular mechanisms in disease treatment (Zhu et al. 2021).

The human gastrointestinal tract harbors more than 10^14^ microorganisms (Zhang et al. 2018). An increasing amount of evidence has highlighted the impact of the gut microbiota on the health and development of diseases (Li et al. 2020). Numerous researchers have identified that gut flora and its derived metabolites are associated with AP (Liu et al. 2025; Pan et al. 2024). In addition, gut microbiota disturbance can trigger metabolite abnormalities, thus affecting the progression and regression of AP (Zhang et al. 2023). Correspondingly, therapeutic strategies based on remodeling the gut flora and then modulating metabolite composition have been emerging consistently in recent years, whereas supplementation with astragalin can augment the potentially beneficial bacteria (e.g., *Ruminococcaceae*) and diminish the harmful bacteria (e.g., *Escherichia-Shigella*) to produce a palliative effect on inflammatory diseases (Peng et al. 2020). Therefore, gut microbiota could be regarded as preferred intervention targets in the application of astragalin against AP.

This study aimed to clarify the pharmacological efficacy of astragalin in the management of AP through in vitro and in vivo experiments and to comprehensively elucidate the underlying pharmacological mechanisms by integrating transcriptomics with a network pharmacology approach. Molecular docking and western blot were used to validate the relevant targets and pathways. Additionally, the effects of astragalin on the gut microbiota composition and metabolites were explored using metagenomic sequencing and untargeted metabolomics, respectively, and its protective effects were investigated using fecal microbiota transplantation (FMT) experiments. Our findings reveal the dual mechanisms of astragalin in gut microbiota modulation and inflammatory pathway regulation, uncovering an innovative intervention strategy for AP.

## Material and methods

### Chemicals and reagents

Caerulein (HY-A0190) and Astragalin (HY-N0015) were purchased from MedChemExpress, USA. Neomycin (N6386) and metronidazole (M1547) were provided by Sigma-Aldrich, USA. Meanwhile, ampicillin (1146GR005) was offered from Biofroxx, Germany, and vancomycin (V301569) was supplied by Aladdin, China.

### Cell culture and induction of the AP cell model

The rat pancreatic acinar cell line AR42J was maintained in F12K medium supplemented with 20% (v/v) fetal bovine serum (FBS) and 1% (v/v) penicillin–streptomycin at 37 °C in a 5% CO₂ humidified incubator. To establish an in vitro model of AP, the cells were stimulated with 100 nM caerulein for 24 h (Song et al. 2022).

### Assessment of cytotoxicity and necrosis

The cytotoxic effect of astragalin toward AR42J cells was determined using the Cell Counting Kit-8 (CCK-8) assay. In brief, cells seeded in 96-well plates were exposed to varying concentrations of astragalin (1, 10, 20 μM) for 24 h, in the presence or absence of caerulein co-stimulation. Subsequently, 10 μL of CCK-8 reagent was added to each well, followed by a 2-h incubation. The absorbance was measured at 450 nm using a microplate reader to determine cell viability. Acinar cell death was further evaluated by live/dead double staining. After treatments, cells were stained with calcein-AM and propidium iodide (PI), and imaged using a fluorescence microscope to distinguish living (green fluorescence) from dying/dead (red fluorescence) cells.

### Analysis of intracellular ROS and apoptosis

After treatments, cells were harvested, washed with cold phosphate-buffered saline (PBS), and processed for the detection of intracellular reactive oxygen species (ROS) and apoptosis. Briefly, for ROS measurement, cells were stained with 10 μM DCFH-DA at 37 °C for 30 min. For apoptosis evaluation, cells were stained with Annexin V-FITC and PI in binding buffer for 15 min. Both procedures were performed in the dark, and fluorescence was analyzed using flow cytometry.

### Mice treatments

Male C57BL/6 J mice (6 weeks, 20–25 g) were supplied by the Department of Zoology, Central South University, and then raised under the following specific-pathogen-free (SPF) environment with a normal diet and regular 12 h of day/light illumination. After a week of acclimatization, 5 randomly assigned groups were as follows: Control (CON) group, Caerulein (CAE) group, CAE + 50 mg/kg Astragalin (CAE + AGL) group, CAE + 75 mg/kg Astragalin (CAE + AGM) group, and CAE + 100 mg/kg Astragalin (CAE + AGH) group, with 5 mice in each group. The dosage of astragalin was selected based on previous studies in the literature (Peng et al. 2020; Tang et al. 2024). During 7 days of intervention, the corresponding concentration of Astragalin (250 μL) was administered by oral gavage, and the other groups were administrated 1% CMC-Na. On day 8, the SAP model was induced in the CAE group and the AG treatment groups by administering intraperitoneally caerulein at a dose of 200 μg/kg in 10 consecutive hourly injections, while the mice in the CON group received similar administration of saline. The body weight, diet, and defecation status were recorded every day. Following the completion of the modeling process, mice were humanely euthanized 12 h after the initial injection. Subsequently, samples of feces, blood, and tissue (pancreas) were obtained and kept at -80 °C. All research procedures were conducted following ARRIVE guidelines and were granted approval and supervision by the Ethics Committee of Central South University (No. FORM-IACUC-01–01).

### Histological examination (H&E) and immunohistochemistry assay (IHC)

Fresh pancreatic tissues were obtained and washed with PBS. After being fixed in a 4% aqueous solution of paraformaldehyde, paraffin embedding and sectioning (5 μm) were performed. A portion of sections was employed for H&E staining and scored for pancreatic histopathological damage via light microscopy (Schmidt et al. 1992). To prepare paraffin sections for IHC experiments, endogenous enzymes and antigens were inactivated or thermally repaired, respectively, followed by blocking and staining with CD68 or Ly6G antibodies. The corresponding secondary antibodies and hematoxylin were then added. After that, the images were captured using a microscope.

### TUNEL assay

After preparation of tissue sections of the pancreas, samples were incubated with Proteinase K solution, followed by washing with PBS twice, then staining with Recombinant TdT Enzyme and 4,6-diamidino-2-phenylindole (DAPI). Next, sections were sealed and photographed through the utilization of an inverted fluorescence microscope.

### Serum biochemical parameters analysis

A procedure of 3,000 rpm, 4 °C for 20 min was set to obtain serum from blood samples. The amylase, lipase, and cytokine levels were assessed using a commercially available kit from ZCIBIO Technology Co., Ltd. (Shanghai, China). The detailed operational procedures followed the guidelines set forth by the manufacturer.

### Network pharmacology

Using “acute pancreatitis” as a keyword, Genecards (https://www.genecards.org/ (relevance score ≥ 5)), OMIM (https://omim.org/), PharmGkb (https://www.pharmgkb.org/), and DrugBank databases (https://go.drugbank.com/drugs/) were used to search for disease targets. To clarify the targets of astragalin, we obtained the chemical structure from the PubChem database (https://pubchem.ncbi.nlm.nih.gov/) and collected astragalin-related targets from Swisstargetprediction (http://www.swisstargetprediction.ch/), pharmMapper (http://www.lilab-ecust.cn/pharmmapper/), and Super-PRED (https://prediction.charite.de/index.php) databases following target validations by the Uniprot database (https://www.uniprot.org/). Then, Venny 2.1 (https://bioinfogp.cnb.csic.es/tools/venny/index.html/) was utilized to acquire the Venn diagram of the astragalin targets and the AP targets. The overlapping targets were imported into the STRING database (https://string-db.org/) to obtain the PPI network and visualize it by Cytoscape 3.10.0. CytoHubba plug-in was chosen to screen for the top 20 hub targets based on the Maximal Clique Centrality (MCC) algorithm.

### Transcriptome analysis

Total pancreatic RNA was required and subsequently subjected to library preparation in accordance with Illumina protocol. The concentration and size distribution of cDNA library were assessed using an Agilent 4200 bioanalyzer, followed by Illumina Novaseq6000 sequencing. High-throughput sequencing was performed as instructed by the manufacturer (Illumina). Then, filtering and mapping to the genome were executed via Seqtk and Hisat2 (version: 2.0.4) respectively (Kim et al. 2015). After that, gene fragments were counted and normalized utilizing stringtie (v1.3.3b) and TMM (trimmed mean of M values) (Mortazavi et al. 2008; Pertea et al. 2016, 2015; Robinson & Oshlack 2010). Subsequently, DEGs were determined, with a particular emphasis on genes exhibiting *P* < 0.05 and |log2FC|> 1 (Benjamini & Yekutieli 2001; Robinson et al. 2010; Robinson & Oshlack 2010).

### KEGG enrichment analysis

To explore the mechanisms of astragalin on AP, KEGG enrichment analysis was performed and visualized through the R software based on the targets in the network pharmacology and transcriptome analysis.

### Molecular docking

To better illustrate the relationship between astragalin and target molecules, we took the intersection of the targets obtained from network pharmacology and transcriptomics. The structures of intersection targets were retrieved from the RCSB Protein Data Bank (RCSB PDB, https://www.rcsb.org/) and were performed pretreatment through PyMOL software. The SDF file of astragalin was downloaded from the PubChem database (https://pubchem.ncbi.nlm.nih.gov/) followed by minimizing energy and transforming to mol2 file through Chem3D22.0.0 software. Finally, AutoDockTools 1.5.6 was utilized to perform “delete water”, “add hydrogens”, “set grid box”, and “molecular docking”. PyMOL software was used to visualize.

### Western blotting

Pancreatic tissue was harvested, quick-frozen, and then homogenized in RIPA buffer. After determining the protein concentration, the protein was denaturized, separated by SDS-PAGE, transferred to a PVDF membrane, and subsequently blocked with 5% skimmed milk for 2 h. Incubated with primary antibodies, including IL-1β (1:1000, Abcam, USA), NLRP3 (1:1000, Servicebio, China), P20-Caspase-1 (1:1000, CST, USA), ASC (1:1000, Bioss, China) overnight at 4 °C, the membrane was then washed and incubated with the corresponding secondary antibody (1:5000, Proteintech, China). The bands were observed by a hypersensitive chemiluminescence kit (Beyotime, China). Relative protein expression was normalized to the β-actin.

### Fecal metagenomics analysis

Following the extraction of DNA using the Fecal Genomic DNA Extraction Kit, sequencing was conducted using an MGISEQ-2000 sequencer and then quality-controlled using KneadData. All effective sequences were annotated and classified using Kraken2 (v2.0.8-beta), and then compared with species sequences in the database to obtain species abundance information at various taxonomic levels. With the help of R software (version 2.15.3), microbial alpha diversity was analyzed using the vegan package, while beta diversity was manifested by the PLSDA and analysis of similarities (ANOSIM). Linear discriminant analysis Effect Size (LEfSe) was employed to identify statistically discriminative microbial taxa among different experimental groups. Functional annotation of metagenomic sequencing was conducted using HUMAnN v3.0.1 and visualized by clustering heatmap.

### Serum untargeted metabolomics profiling

Serum untargeted metabolome analysis was conducted based on isolated metabolites using a Vanquish ultra-performance liquid chromatography system (Thermo Fisher Scientific). After conversion to mzXML format of raw data by ProteoWizard software and a series of data processing, the metabolites were annotated according to the BiotreeDB (V2.1) database. SIMCA16.0.2 was used to conduct principal component analysis (PCA). Additionally, Variable Importance in the Projection (VIP) > 1 and *P* < 0.05 were considered significantly differential metabolites and presented in volcano plots.

### FMT

Donor mice were randomly assigned to the Control donor group (CON-D group), AP donor group (CAE-D group), and AG donor group (AGH-D group). The mice were administered following the above experimental methods. In parallel, recipient mice were randomly assigned into the CON receptor group (CON-R group), CAE receptor group (CAE-R group), and AG receptor group (AG-R group). Before the FMT experiment, recipient mice were administered 200 μL of quadruple antibiotics in advance (vancomycin 0.5 mg/mL, neomycin 1 mg/mL, ampicillin 1 mg/mL, and metronidazole 1 mg/mL) by oral gavage for 1 week. Fresh feces (250 mg) were collected from the donor group on day 8, homogenized with sterile saline (1 mL) by vigorous vortexing, and then centrifuged at 600 g for 3 min. After that, the supernatant was aspirated and subjected to centrifugation. As a result of discarding the supernatant and mixing it with saline (1 mL), the bacterial suspension was acquired and thereafter transplanted to recipient mice via gavage (200 μL) for 7 days (Ba et al. 2017). On day 8, the SAP animal model was induced in the CAE-R and AG-R groups. After modeling, these three groups were named by the FMT + Con group, FMT + AP group, and FMTAGH + AP group, respectively. Relatively regular information was assessed and noted. After dissection, pancreatic tissue and blood were obtained and stored at -80 °C.

### In vivo safety assessment

Ten mice were randomly allocated into two groups: one group received 100 mg/kg astragalin (AG-H group) via oral gavage for 7 consecutive days, while the Control group was administered an equivalent volume of 1% CMC-Na. On day 8, all mice were euthanized, and major organs (heart, liver, spleen, lung, kidney, and pancreas) were harvested for H&E staining. Body weights were measured and recorded at 48-h intervals using a calibrated electronic balance (precision ± 0.1 g). Serum samples, including ALT, AST, Cr, and BUN, were collected to assess hepatic and renal function markers using an automatic biochemical analyzer.

### Statistical analysis

All data were analyzed using GraphPad Prism 9.0 software. To investigate the statistical difference between two independent samples, a Student's t-test or a Mann–Whitney U test was performed. In the comparison of multiple groups, a one-way analysis of variance (ANOVA) was utilized, followed by Dunnett's t-test, while a Kruskal–Wallis test was used for a nonparametric test. Spearman correlation analysis was determined by *P*-value and correlation coefficient (*P* < 0.05, correlation coefficient > 0.8 as a threshold). Differences *P* < 0.05 were considered statistically significant.

## Results

### Astragalin attenuated caerulein-induced injury in AR42J cells

**Fig. 1:**
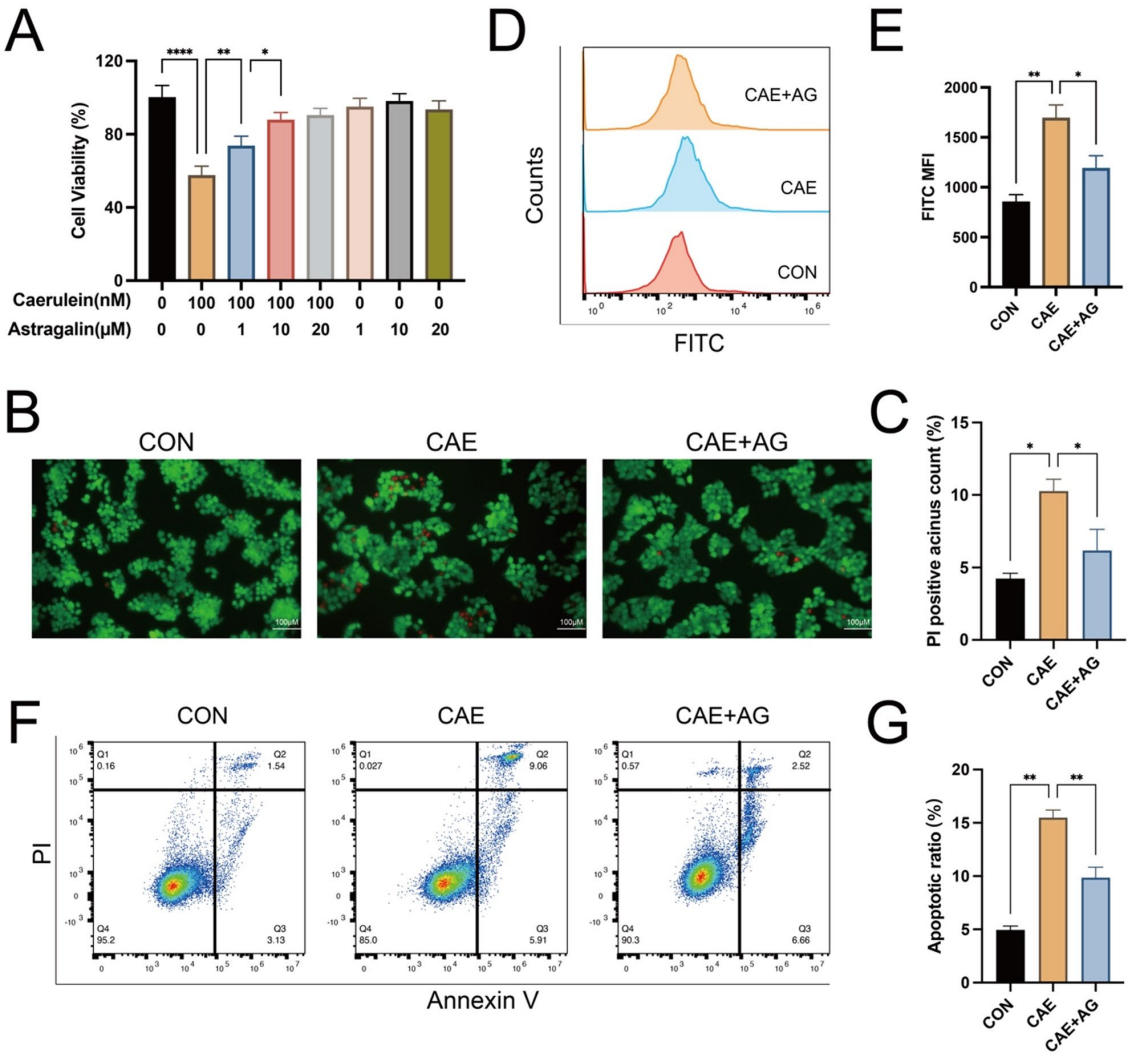
Effect of astragalin administration in caerulein-induced AP in AR42J cells. (**A**) CCK8 assay of astragalin on AR42J cells. (**B** and** C**) Live/dead staining showing living (green) and dying/dead (red) cells. (**D** and** E**) Intracellular ROS levels detected by DCFH-DA fluorescence via flow cytometry. (**F** and** G**) Apoptotic rate analyzed by Annexin V-FITC/PI staining and flow cytometry. Data are expressed as mean ± standard deviation (SD) of independent experiments. N = 3. *P < 0.05, ** *P* < 0.01, ****P < 0.0001

The CCK-8 assay indicated that astragalin (1, 10, 20 μM) alone exhibited no adverse effects but significantly restored cell viability in caerulein-induced AR42J cells, with 10 μM selected as optimal due to comparable efficacy to 20 μM (Fig. 1A). Evaluation of the pharmacological activity against acinar cell necrosis was corroborated and visualized by live/dead staining, which showed a remarkable reduction in PI-positive cells at this optimal concentration (Fig. 1B, C). Further investigation revealed that 10 μM astragalin effectively attenuated intracellular ROS accumulation (Fig. 1D, E) and significantly reduced the apoptotic rate in caerulein-stimulated AR42J cells (Fig. 1F, G).

### Astragalin ameliorated pancreatic injury and inflammation in caerulein-induced AP mice

**Fig. 2:**
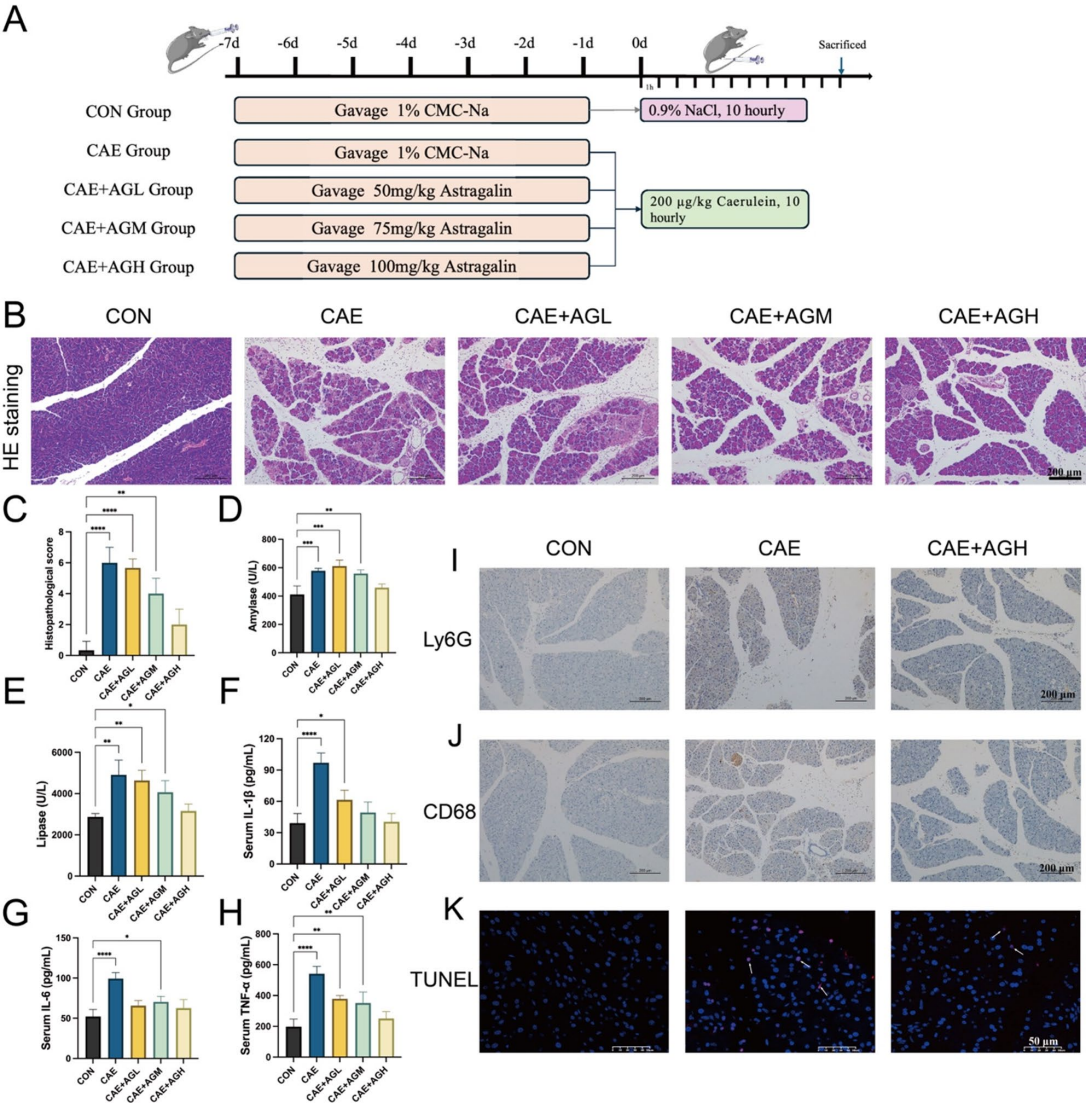
Effect of astragalin treatment at different doses in caerulein-induced AP. (**A**) Schematic diagram of experimental design. (**B**) Images of H&E staining in pancreatic tissue. (**C**) Histopathological scores. (**D** and** E**) Serum amylase and lipase. (**F**–**H**) Serum inflammatory cytokines. (**I** and** J**) Immunohistochemistry of Ly6G and CD68 in pancreatic tissues. (**K**) TUNEL staining. Data are expressed as mean ± standard deviation (SD) of independent experiments. N = 5. *P < 0.05, ** *P* < 0.01, ***P < 0.001, ****P < 0.0001 (versus the CON group)

Building upon the promising efficacy of astragalin observed in our in vitro AP model, we therefore extended the investigation to an AP mouse model, employing it to elucidate its therapeutic effects and underlying mechanisms (Fig. 2A). The mice were split up into five groups: CON group, CAE group, CAE + AGL group, CAE + AGM group, and CAE + AGH group supplemented with varying intervention doses of astragalin. Pancreatic histopathological injury (Fig. 2B, C), amylase (Fig. 2D), lipase (Fig. 2E), and inflammatory cytokines (Fig. 2F-H) levels were markedly upregulated in the CAE group in comparison to the CON group, whereas pancreatic edema, inflammation, and necrosis were alleviated to varying degrees in the AG intervention groups. In conclusion, no notable discrepancy was observed in the pancreatic pathological scores between the CON group and the CAE + AGH group (Fig. 2C). Similar to that, no noteworthy alterations were observed in the amylase (Fig. 2D), lipase (Fig. 2E), inflammatory cytokines (Fig. 2F-H) in the CAE + AGH group in contrast to the CON group. The above results demonstrated that 100 mg/kg/d astragalin could improve pancreatic injury and systemic inflammatory response. In addition, inflammatory cell infiltration and apoptosis were further examined by IHC and TUNEL assay as a result of reduced Ly6G + neutrophils (Fig. 2I), CD68 macrophages (Fig. 2J), and TUNEL-positive cells (Fig. 2K) in pancreatic tissues under astragalin administration.

### Target collection and PPI network of astragalin on AP

**Fig. 3:**
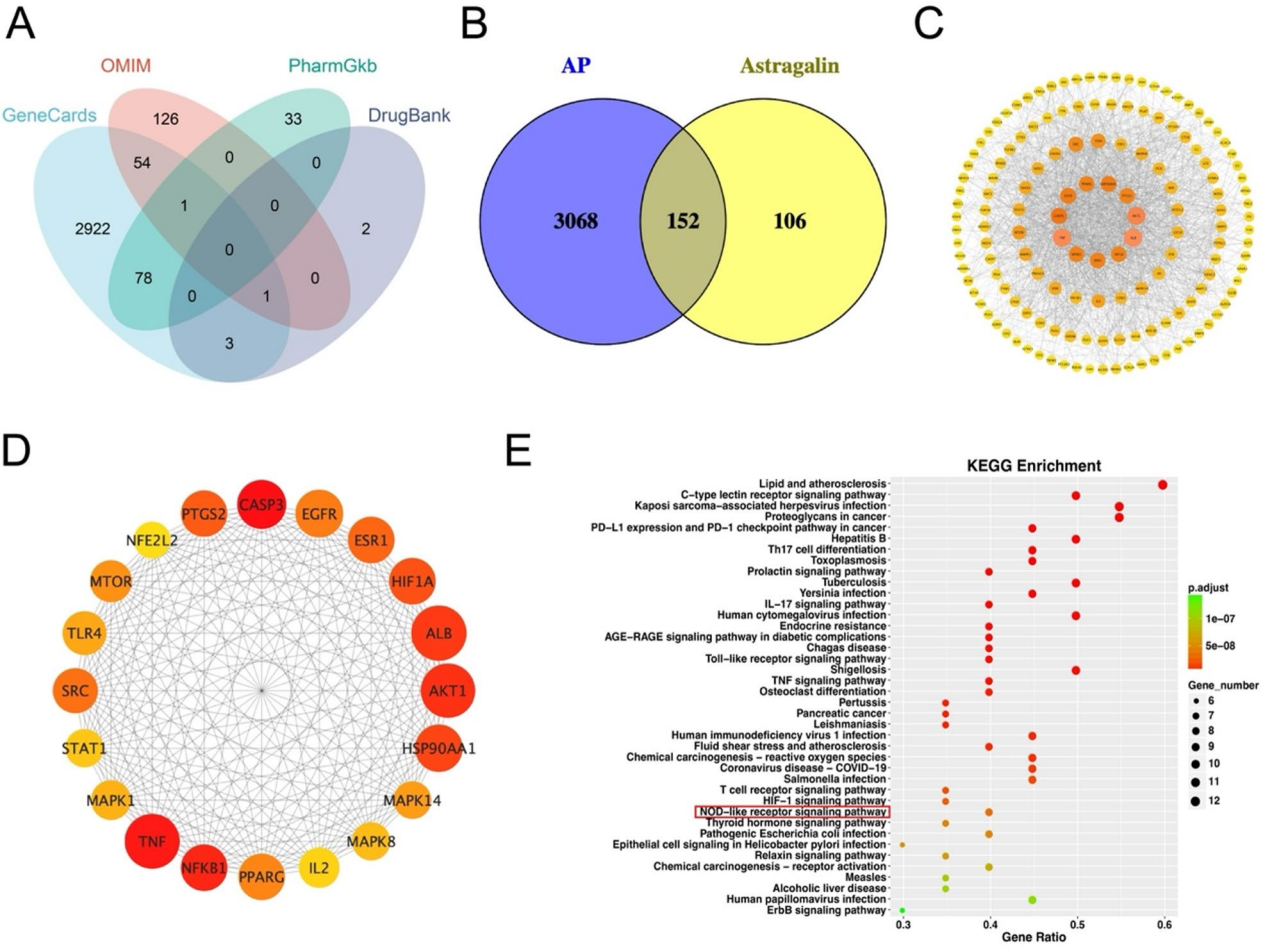
The network pharmacology analysis of astragalin on AP. (**A**) Venn diagram of AP targets. (**B**) Venn diagram of overlapping targets with astragalin in AP. (**C**) PPI network. (**D**) Core network of the top 20 core targets of astragalin for AP. (**E**) KEGG-enriched bubble plot

We obtained a total of 3220 AP targets from 4 public databases, including 3059 targets from the GeneCards database, 183 targets from the OMIM database, 112 targets from the PharmGkb database, and 6 targets from the DrugBank database (Table S1, Fig. 3A). Astragalin-related targets were collected by integrating Swisstargetprediction, PharmMapper, and Super-PRED databases (Table S2). A Venn diagram was drawn to acquire the 152 overlapping targets, as shown in Fig. 3B. The above 152 overlapping targets were imported into the STRING database. Network topology analysis was performed through Cytoscape software. Then the PPI network was obtained (Fig. 3C) and the MCC algorithm in the CytoHubba plug-in was employed to identify the top 20 hub genes (Fig. 3D). Moreover, KEGG analysis revealed the main enrichment in NOD-like receptor (NLRP) signaling pathway (Fig. 3E).

### Analysis of pancreatic transcriptomics in caerulein-induced AP

**Fig. 4:**
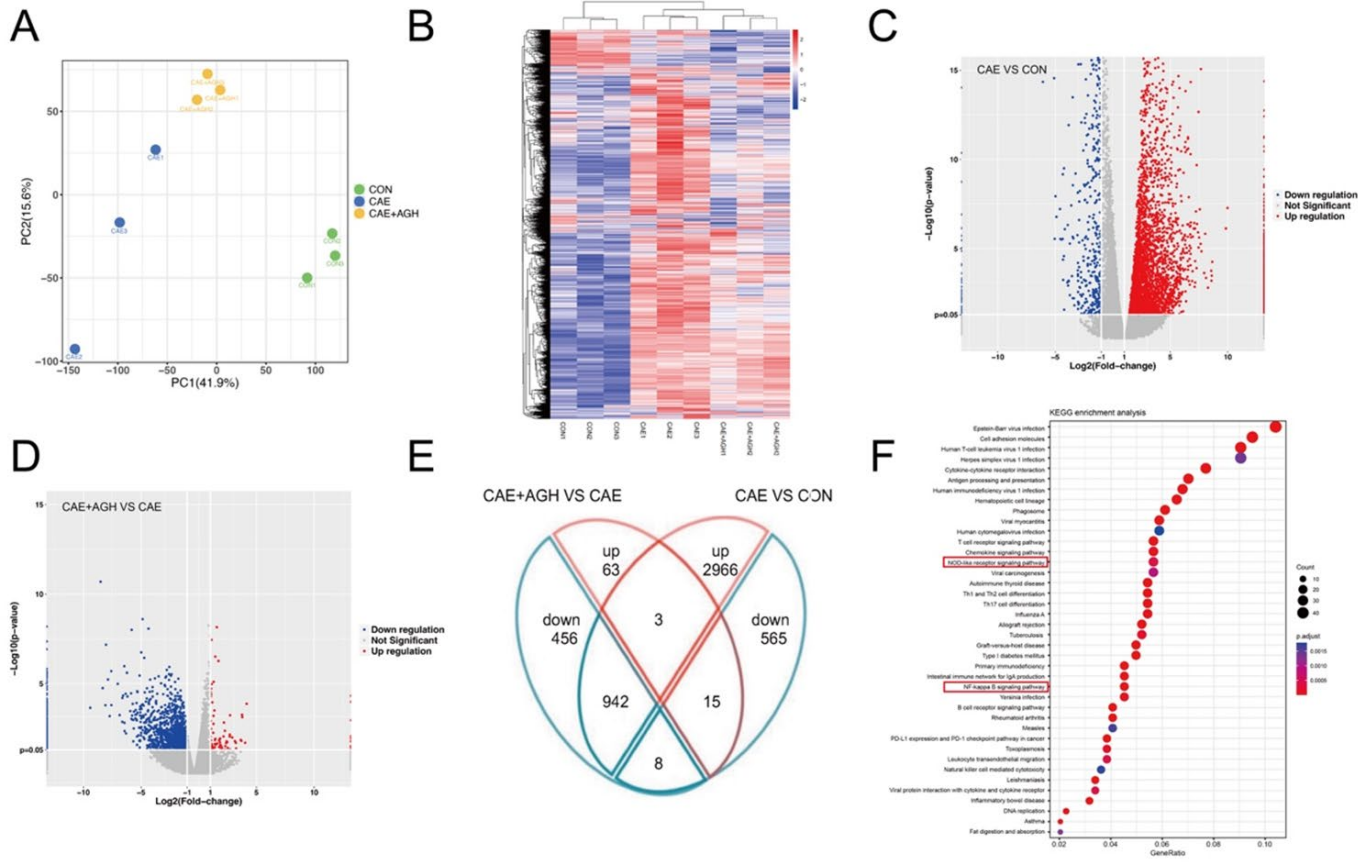
Astragalin partially reversed pancreatic tissue mRNA alterations in caerulein-induced AP mice. (**A**) PCA plot to visualize global transcriptomic profiles among the CON, CAE, and CAE + AGH groups. (**B**) Clustering heatmap of DEGs. (**C**) Volcano plot between CON and CAE groups. (**D**) Volcano plot between CAE and CAE + AGH groups. (**E**) Venn plots of differential genes at the intersections among groups. (**F**) KEGG-enriched bubble plot. N = 3

To validate the intrinsic mechanism of astragalin against AP obtained from network pharmacology analysis, RNA sequencing was performed. PCA visualization and clustering heatmap of DEGs showed distinct clustering patterns in global transcriptomic profiles among the CON, CAE, and CAE + AGH groups (Fig. 4A, B). As illustrated in Fig. 4(C, D), differentially expressed 15 down-regulated genes and 942 up-regulated genes were reversed after astragalin administration (Fig. 4E). Besides, the results of KEGG indicated the main enrichment in the NF-κB signaling pathway and NOD-like receptor signaling pathway (Fig. 4F).

### Comprehensive analysis of network pharmacology and transcriptomics

Intersecting 152 targets acquired from network pharmacology with 957 DEGs from transcriptome yielded 12 target genes (DUSP6, TOP2A, RAC2, SYK, STAT1, CD38, LCK, XDH, CCNA2, PLK1, ITK, DPP4). Intersecting relative enrichment pathways, the NLRP inflammatory pathway was highlighted in the regulation of AP by astragalin.

### Astragalin alleviated AP via pathways involving NLRP inflammatory signaling

**Fig. 5:**
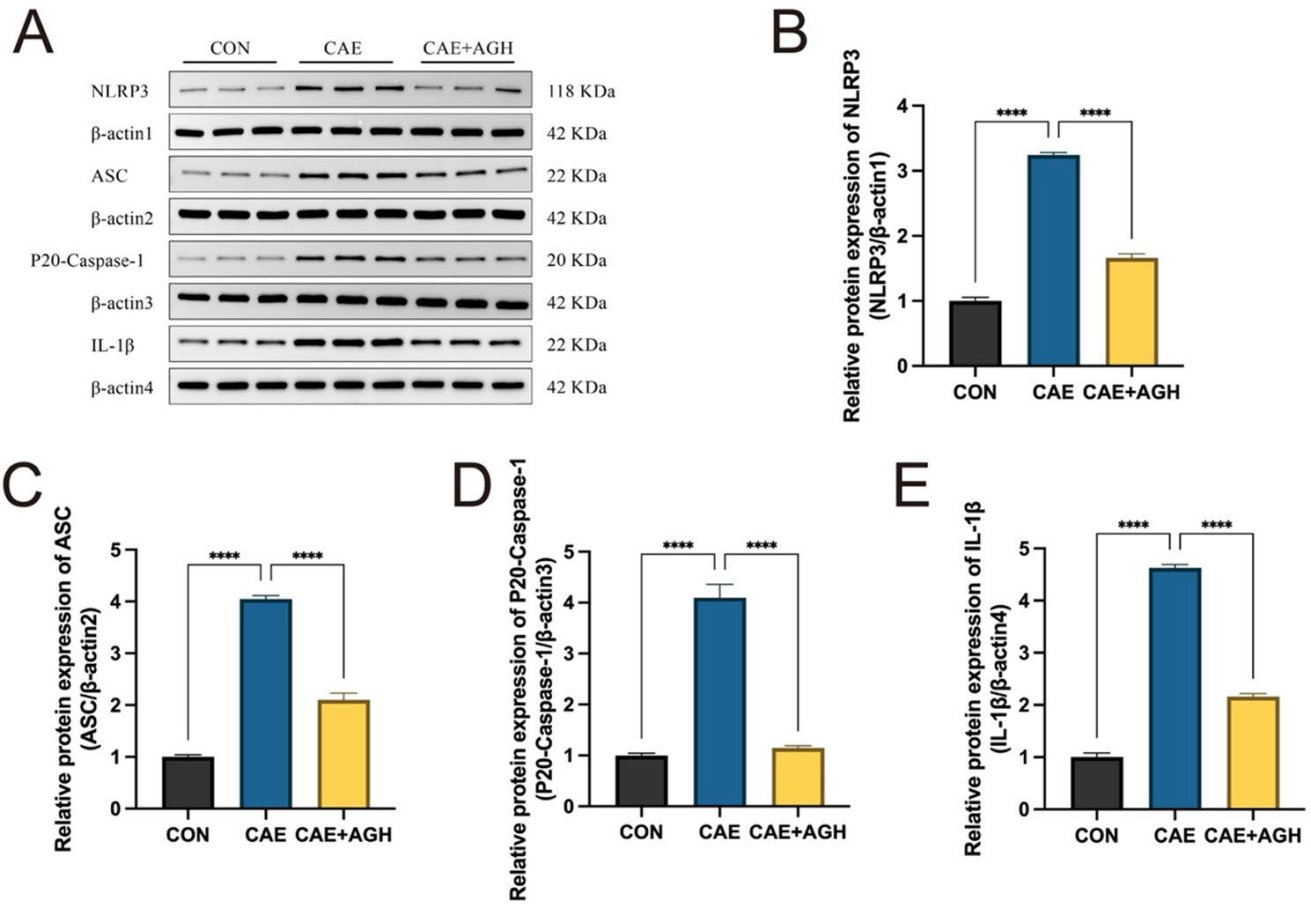
Astragalin alleviated AP via pathways involving NLRP3 signaling. (**A**–**E**) Western blot of the NLRP3 signaling pathway. The uncropped blots are shown in the Original Western blot image file. Data are expressed as mean ± SD of independent experiments. N = 3. ****P < 0.0001 (versus the CAE group)

NLRP3 signaling pathway has attracted considerable attention due to its critical role in inflammatory processes, which are pivotal for the progression of AP (Zhao et al. 2024). In this study, the astragalin treatment markedly reduced the expression of NLRP3, P20-Caspase-1, ASC, and IL-1β proteins in the pancreatic tissue, whereas they were dramatically enhanced in the CAE group (Fig. 5), suggesting that activation of NLRP3 was manifested in AP, and astragalin administration had a depressant effect.

### Molecular docking

**Fig. 6:**
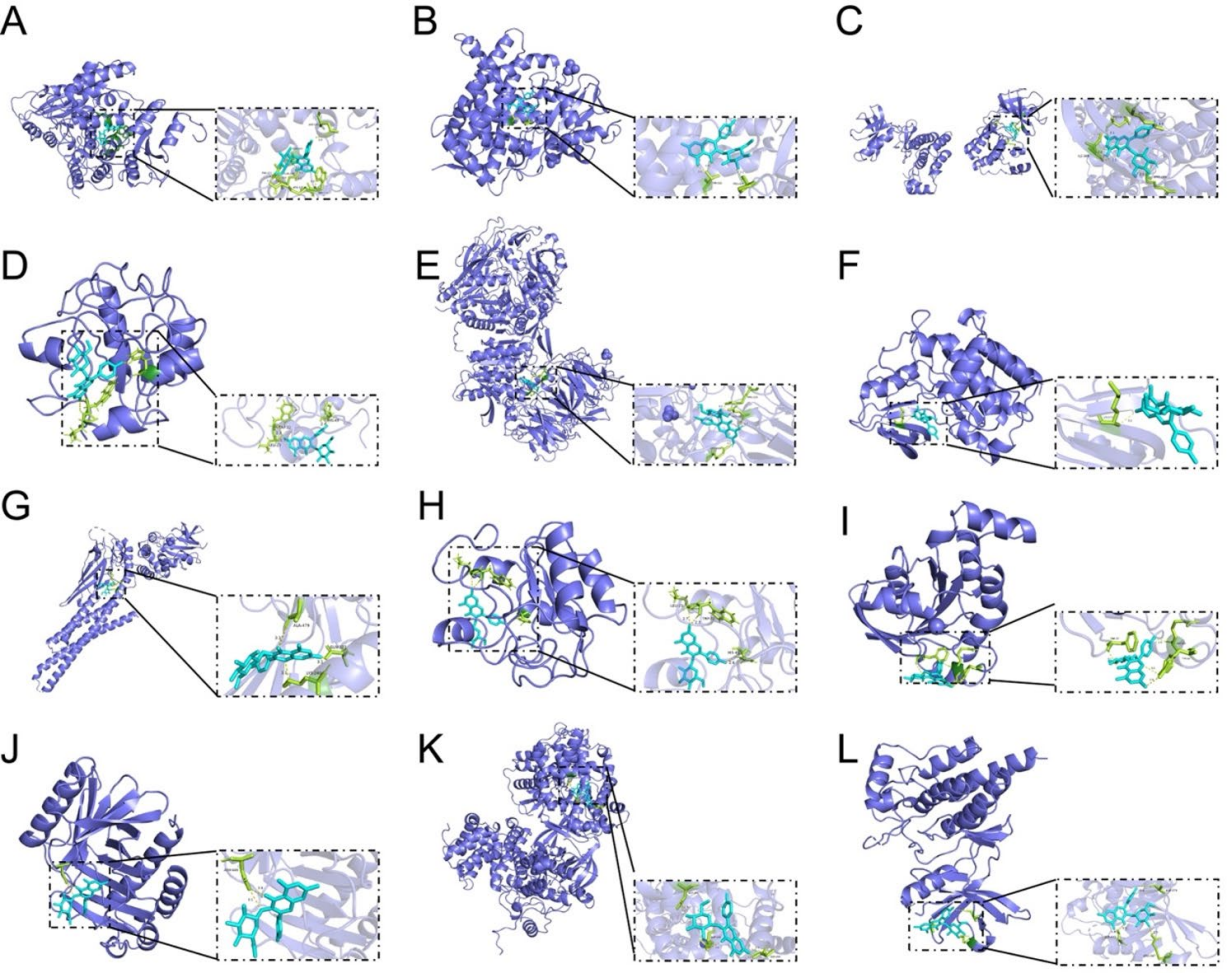
Molecular docking visualization results. (**A**) CD38. (**B**) XDH. (**C**) ITK. (**D**) TOP2A. (**E**) DPP4. (**F**) LCK. (**G**) STATA1. (**H**) DUSP6. (**I**) RAC2. (**J**) PLK1. (**K**) CCNA2. (**L**) SYK

Based on comprehensive network pharmacology and transcriptomics analysis, we chose the 12 target genes as the receptors and astragalin as the ligand to perform molecular docking. It was noted that all the binding energies were <  − 4.25 kcal/mol, which was regarded to have a good binding affinity (Table 1). The specific visualization results were shown in Fig. 6. The above results validated the accuracy of network pharmacology and transcriptomics.

**Table 1:** Molecular docking score

PDB ID	Compound	Molecular Docking Score (kcal/mol)
2o3q	CD38	-9.3
3eqm	XDH	-9.0
3qgy	ITK	-8.9
1zxm	TOP2A	-8.5
4a5s	DPP4	-8.3
2of2	LCK	-8.2
7nuf	STAT1	-7.7
1hzm	DUSP6	-7.3
2w2t	RAC2	-7.3
5nfu	PLK1	-7.0
4eoj	CCNA2	-5.5
6ssb	SYK	-4.7

### Astragalin influenced the microbial diversity in caerulein-induced AP mice

To investigate the impact of astragalin on gut microbiota composition in AP mice, metagenomic sequencing was performed on DNA extracted from fecal samples collected from the CON, CAE, and CAE + AGH groups. The species accumulation curve confirmed an adequate sampling size and robust data reliability (Fig. S1A). In terms of αlpha diversity, a reflection of the diversity and abundance of species was assessed. There was a substantial decline in the Chao 1, Shannon, and Simpson indexes in the CAE group compared to the CON group, while these three indexes showed no statistical significance in the CAE + AGH group (Fig. S1B-D). This result demonstrated that astragalin reversed the α-diversity of gut microbiota in the CAE group. Next, PLSDA and ANOSIM analyses were utilized to reflect the β-diversity. As shown in Fig. S1(E, F), there were clear separations among the CON, CAE, and CAE + AGH groups, suggesting a remarkable difference in the structure of the species composition. In conclusion, astragalin intervention resulted in a modification of the microbial structure in AP.

### Astragalin modified the compositions of gut microbiota in caerulein-induced AP

**Fig. 7:**
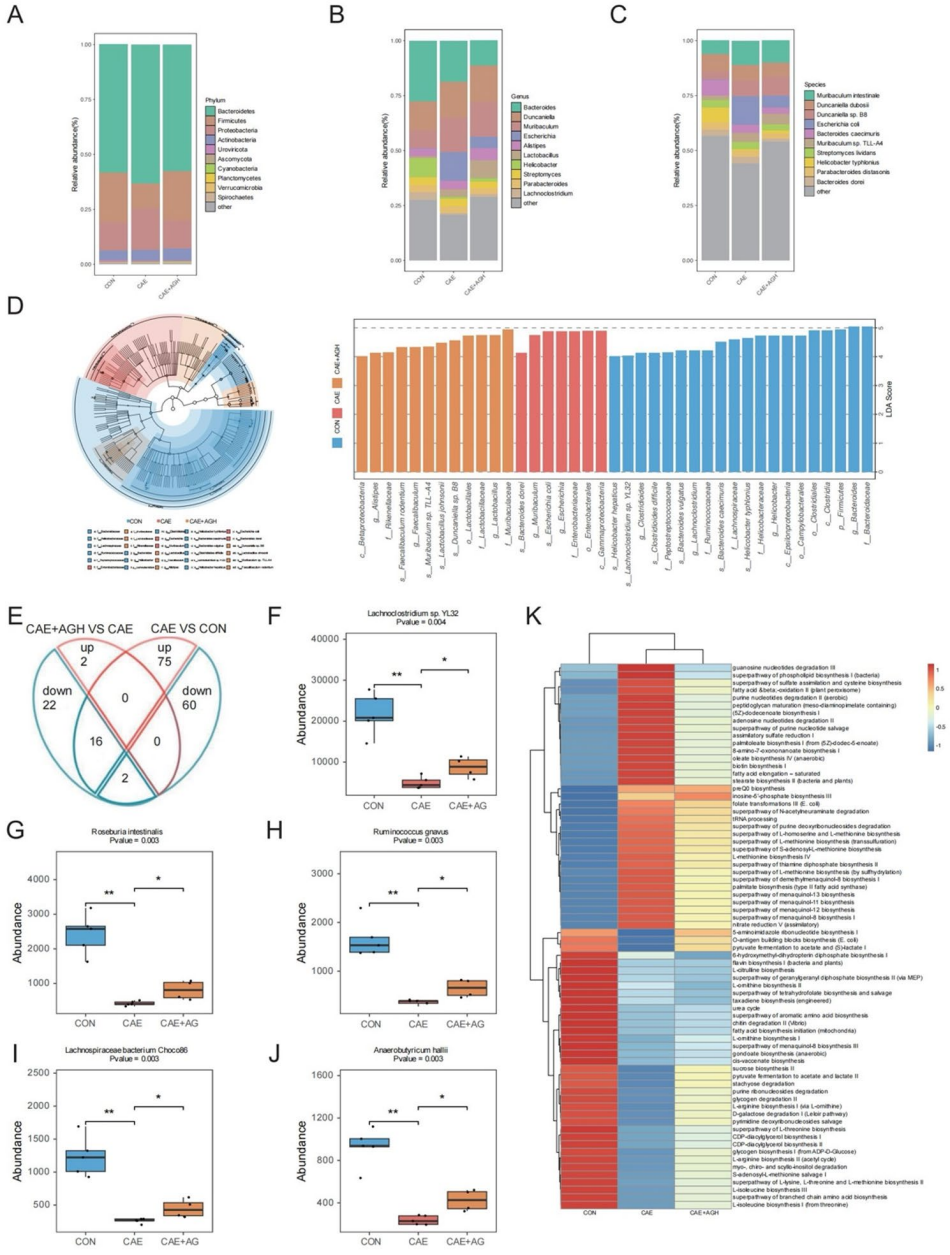
Effects of astragalin on the composition of gut microbiota. (**A**) Abundance of the intestinal microbiota at the phylum level. (**B**) Abundance of the intestinal microbiota at the genus level. (**C**) Abundance of the intestinal microbiota at the species level. (**D**) Taxonomic cladogram and distribution histograms derived from the LEfSe analysis. (**E**) Venn plot showed the reversed strains in AP supplemented with astragalin. (**F**–**J**) The abundance comparison of representative reversed species among three groups, (**F**) *Lachnoclostridium* sp. YL32, (**G**) *Roseburia intestinalis*, (**H**) *Ruminococcus gnavus*, (**I**) *Lachnospiraceae bacterium* Choco86, and (**J**) *Anaerobutyricum hallii*. (**K**) Functional annotation clustering heatmap. Data are expressed as mean ± SD of independent experiments. N = 4–5

Next, we performed comparative analysis of relative abundances of dominant taxa at the phylum level (Fig. 7A). Compared with the CON group, the CAE group exhibited increased abundance of *Bacteroidetes* and *Proteobacteria*, but decreased levels of *Firmicutes*. Astragalin intervention effectively restored the abundance of *Firmicutes* and decreased the abundance of *Bacteroidetes* and *Proteobacteria*. Also, astragalin reversed the gut microbiota at the genus and species levels in AP, as shown in Fig. 7(B, C). The LEfSe analysis was employed to identify microbial biomarkers with significant differences in abundance among groups. As depicted in Fig. 7D, the LEfSe analysis identified distinct microbial signatures among the CON, CAE, and CAE + AGH groups (LDA score > 4, *P* < 0.05), with *Muribaculum* and *Escherichia* significantly enriched in the CAE group. At the same time, *Alistipes*, *Faecalibaculum*, and *Lactobacillus* were predominant in the CAE + AGH group. To further visualize the species-level compositional differences among the three groups, a Venn diagram graphically summarized that astragalin significantly reversed 16 bacterial species, which were markedly downregulated after AP induction (Fig. 7E). The abundance comparison of representative species is presented in Fig. 7F-J.

Additionally, functional annotation of metagenomic sequencing elucidated the role of astragalin (Fig. 7K). The results demonstrated elevated purine nucleotide degradation, fatty acid β-oxidation, superpathway of phospholipid biosynthesis I (bacteria), and peptidoglycan maturation (meso-diaminopimelate containing) in the CAE group, which were attenuated in the CAE + AGH group. Conversely, pyruvate fermentation to acetate and lactate and amino acid biosynthesis-related functions (including L-arginine, L-threonine, S-adenosyl-L-methionine, L-lysine, L-methionine, L-isoleucine, branched-chain amino acids, and L-isoleucine) were significantly impaired in the CAE group but were restored following astragalin treatment.

These findings collectively suggested that astragalin not only reversed caerulein-induced gut microbiota dysbiosis in AP mice, but also optimized microbial functionality by modulating specific metabolic pathways, thereby exerting beneficial effects on AP pathophysiology.

### Astragalin reshaped metabolic profiles and modulated the gut microbiota-metabolite-gene network in caerulein-induced acute pancreatitis

**Fig. 8:**
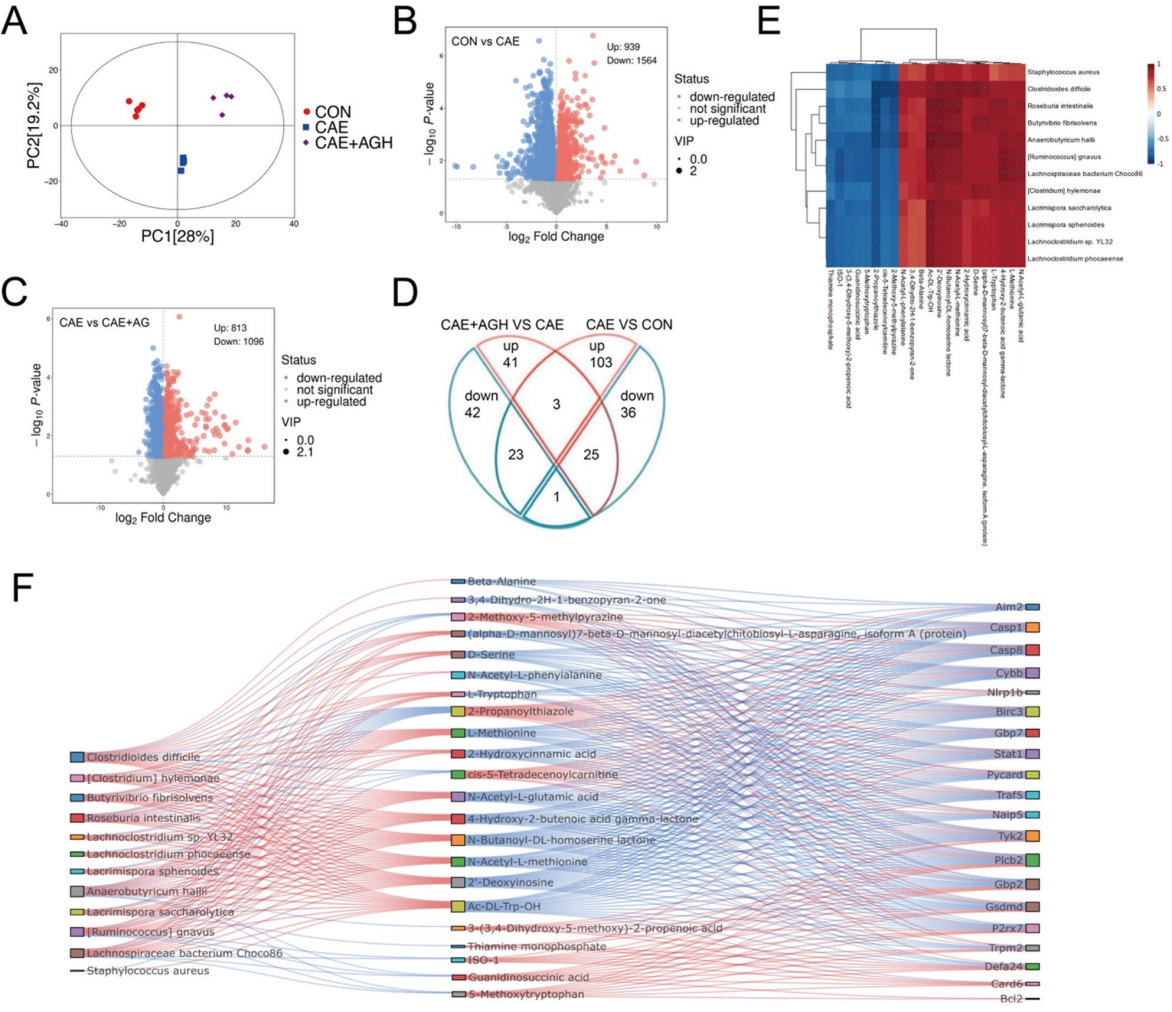
Astragalin reshaped metabolic profiles and regulated the gut microbiota-metabolite-gene network in AP.(A) PCA plot. (B) Volcano plot between CON and CAE groups. (C) Volcano plot between CAE and CAE+AGH groups. (D) Venn plot showed the reversed metabolites in AP supplemented with astragalin. (E) Gut microbiota-metabolite correlation heatmap. (F) Gut microbiota-metabolite-gene network. N=4-5.

Following the established effects of astragalin on the NLRP3 pathway and gut microbiota, we conducted an untargeted metabolomics analysis to explore its downstream mechanisms mediated by the gut microbiota and their connection with NLRP3 signaling. PCA plot revealed a clear separation among the CON, CAE, and CAE + AGH groups, indicating distinct metabolic profiles (Fig. 8A). Volcano plots further illustrated the expression trends of metabolites across comparison groups (Fig. 8B). To clarify the relationship among astragalin-effective gut microbiota and serum metabolites, the metabolites regulated by astragalin were screened, and the results demonstrated that astragalin administration markedly upregulated 23 metabolites that changed in AP, as well as down-regulated 25 metabolites that altered in AP (Fig. 8D). As a result of further Spearman correlation analysis on the 16 strains and 48 metabolites with the screening criterion of the absolute value of correlation coefficient > 0.8, gut microbiota-metabolite correlation heatmap was constructed with *Lachnoclostridium* sp. YL32, *Roseburia intestinalis*, *Ruminococcus gnavus*, *Lachnospiraceae bacterium* Choco86, *Anaerobutyricum hallii*, etc. as the main core strains, and 22 metabolites, including 5-Methoxytryptophan, D-Serine, L-Tryptophan, L-Methionine, etc. as the core metabolites (Fig. 8E).

Subsequently, the 22 serum metabolites were subjected to Spearman correlation analysis with 20 NLRP3 pathway-related genes (4 genes were excluded as they did not meet the screening criteria of *P* < 0.05 and correlation coefficient > 0.8) that were previously identified via transcriptomic sequencing as modulated by astragalin. By integrating these results with differential microbiota-metabolite correlation network, we mapped a differential microbiota-metabolite-gene network and visualized it using a Sankey diagram (Fig. 8F).

### Fecal microbiota transplantation mitigated caerulein-induced AP

To further verify the role of gut microbiota in astragalin treatment, mice underwent microbiota clearance using a classical quadruple antibiotic combination regimen, and then the feces of mice after astragalin administration were transplanted and modeled for comparative observation of the therapeutic effects (Fig. S2A). HE staining demonstrated that, compared with the FMT + CON group, the pathological changes of inflammation and necrosis were manifested in the FMT + AP group, whereas those were alleviated in the FMTAGH + AP group (Fig. S2B). Furthermore, the measurement of enzymes and inflammatory cytokines was significantly elevated in the FMT + AP group, while experiencing a decline in the FMTAGH + AP group (Fig. S2C-G). Taken together, the above results reasonably demonstrated that gut microbiota were indispensable and prominent for the efficacy of astragalin against AP.

### Astragalin showed no significant toxicity in vivo

To evaluate the clinical translation potential of astragalin, we conducted systematic safety assessments. Results demonstrated that astragalin caused no histopathological damage to vital organs (Fig. S3A), elicited no hepatic or renal toxicity (Fig. S3B-E), and induced no adverse effects on body weight (Fig. S3F), indicating favorable safety profiles.

## Discussion

In our current study, we explored the novel therapeutic direction behind AG against AP. Initially, astragalin demonstrated promising efficacy in an in vitro AP cell model. On this basis, to further investigate in vivo effects and underlying mechanisms, an AP mouse model was successfully constructed by intraperitoneal injection of high-dose caerulein (Gao et al. 2021; Steele et al. 2015). Firstly, it was discovered that astragalin enhanced pathological injury of the pancreas and concurrently decreased serum enzyme levels during AP. Subsequently, 100 mg/kg AG was identified to be the optimal therapeutic dose, which significantly ameliorated the inflammatory response and pancreatic apoptosis. Furthermore, we integrated network pharmacology and transcriptomic analyses to explore potential mechanisms, followed by validation through molecular docking and western blot. Next, metagenomic and serum metabolomic profiles in AP mice were shown to be partially reversed following astragalin treatment. Subsequently, correlation analysis was performed to establish an astragalin-effective network linking gut microbiota, metabolites, and genes. Lastly, the FMT experiment was performed to validate that the therapeutic effect of astragalin against AP mice was partly dependent on the gut microbiota. In summary, astragalin ameliorated AP through a multifaceted mechanism involving the regulation of the inflammatory pathway and remodeling of gut microbiota composition, which were interconnected.

Several studies have indicated that astragalin exerts anti-inflammatory properties through its impact on inflammatory signaling pathways. For instance, astragalin was observed to mitigate DSS-induced colitis by inhibiting NF-κB (Peng et al. 2020). Besides, astragalin could alleviate inflammatory osteolysis by downregulating the ROS and MAPK signaling pathway (Xing et al. 2022). Therefore, astragalin holds great potential as an agent for AP treatment. However, its effects and molecular mechanisms require further exploration, which hampers its clinical application. To further explore astragalin’s mechanism of action, we integrated network pharmacology and transcriptomics analysis and found that astragalin mainly affected the NLRP signaling pathways in AP. Many proteins within the NLR family have been recognized as key parts of the innate immune system, and NLRP3 is the most intensively investigated inflammasome-associated receptor. The NLRP3 inflammasome is known to be activated in AP, with activation level correlating with the severity of AP (Jia et al. 2020; Sendler et al. 2020). Also, it was demonstrated that exosomes derived from plasma could induce pancreatitis-related lung injury by activating NLRP3-dependent focal death in macrophages within the alveoli (Wu et al. 2022). The present study not only verified the critical role of NLRP3 signaling in AP progression but also revealed its targeted inhibition by astragalin. In light of the consistency of network pharmacology, transcriptomics analysis, and aforementioned western blot results, it can be postulated that astragalin may exert an anti-inflammatory effect in AP mice via the NLRP3 signaling pathway.

Pharmacological studies have demonstrated that astragalin is poorly absorbed orally (Etxeberria et al. 2013), and most of the components reach the colon without digestion and then are metabolized by microorganisms. This study demonstrated that the gut microbiota of AP exhibited notable alterations under the administration of astragalin with an elevated diversity of gut microbiota, which was similar to another study of colitis (Peng et al. 2020). Regarding gut microbiota composition, compared with the CON group, the CAE group exhibited increased abundances of *Bacteroidetes* and *Proteobacteria* and decreased *Firmicutes* abundance, which were restored by astragalin treatment. In a previous study, Amuc_1100 pretreatment was shown to alleviate AP by reducing the proportions of *Bacteroidetes* and *Proteobacteria* while increasing *Firmicutes* abundance (Wang et al. 2024), which aligns with our findings.

At the genus level, it is noteworthy that astragalin supplementation significantly enriched beneficial bacterial genera (including *Alistipes*, *Faecalibaculum*, and *Lactobacillus*) while suppressing potential pathogenic genera such as *Muribaculum* and *Escherichia*, thereby modulating gut microbiota composition. *Alistipes* is predominantly found in healthy individuals(Shkoporov et al. 2015), and has been associated with the production of acetate and propionate, exhibiting potential anti-inflammatory properties(Wang et al. 2023). Notably, its abundance shows an inverse correlation with pro-inflammatory cytokine levels (Fang et al. 2023). Short chain fatty acids (SCFAs, mainly consisting of acetate, propionate, and butyrate), as key microbial metabolites, contribute to AP remission through remodeling gut microbiota composition, strengthening the intestinal epithelial barrier, and modulating inflammatory responses (Yan et al. 2023). *Faecalibaculum*, an emerging beneficial bacterial genus, functions as another SCFA producer. Current evidence indicates that *Faecalibaculum* exerts anti-inflammatory effects by stimulating the expansion of colonic regulatory T cells (Zagato et al. 2020). Meng et al. further demonstrated in an acute kidney injury model that *Faecalibaculum* abundance positively correlated with intestinal barrier markers, including ZO-1, OCLN, and MUC-2 (Meng et al. 2024). Although direct evidence linking *Alistipes* and *Faecalibaculum* to AP is currently lacking, its demonstrated anti-inflammatory and gut barrier-protective properties suggest considerable therapeutic potential for AP management. As for *Lactobacillus*, Zhou et al. observed significant downregulation of *Lactobacillus* in an AP rat model, and further studies revealed that its metabolite norharman could alleviate AP progression by inhibiting histone deacetylase activity, enhancing H3K9/14 acetylation of Rftn1, and consequently suppressing M1 macrophage activation (Zhou et al. 2023). Moreover, complementary findings from TLR4-deficient mouse models demonstrated that *Lactobacillus* protects against AP-associated intestinal injury through NOD2-dependent promotion of Paneth cell function (Qi-Xiang et al. 2022).

Shotgun metagenomic analysis in this study provided species-level resolution of microbiome alterations, offering greater taxonomic and functional insight compared to 16S rRNA profiling. Astragalin treatment significantly restored the abundance of key bacterial species, including *Lachnoclostridium* sp. YL32, *Roseburia intestinalis*, *Ruminococcus gnavus*, *Lachnospiraceae bacterium* Choco86, and *Anaerobutyricum hallii*. *Roseburia intestinalis* and *Anaerobutyricum hallii* are established butyrate-producing species with demonstrated anti-inflammatory, antioxidant, and immunomodulatory properties (Deyaert et al. 2024; Han et al. 2024; Shen et al. 2022; Tintore et al. 2024). While the functional role of *Lachnoclostridium* sp. YL32 remains incompletely characterized, emerging evidence suggests its potential as a beneficial commensal, with disease-protective effects linked to abundance modulation (Li et al. 2024). *Ruminococcus gnavus and Lachnospiraceae bacterium* Choco86 are both members of the *Lachnospiraceae* family. Bacteria within the Lachnospiraceae have been reported to degrade complex polysaccharides into SCFAs, which serve as crucial energy sources for the host (Lee et al. 2018). Furthermore, as potential butyrate producers (Louis & Flint 2017), they may play significant roles in the context of AP. However, their specific functions in AP remain unexplored, warranting further investigation. Furthermore, astragalin administration significantly altered pivotal gut microbial metabolic pathways, notably amino acid, carbohydrate, and lipid metabolism, which aligns with our previous study (Jia et al. 2024). Subsequently, metabolomic and correlation analyses identified 22 core metabolites, most of which are substrates or derivatives involved in the aforementioned metabolic pathways. Although these metabolites have not been specifically reported in AP, they have been documented in other inflammatory diseases to exert therapeutic effects, highlighting their potential relevance in AP management. However, the specific roles of these metabolites in AP, as well as their targeting within the astragalin-effective gut microbiota-metabolite-gene network, require further experimental validation.

In this study, the results systematically elucidated the effect and intrinsic mechanism of astragalin in treating AP by combining multi-omics and network pharmacology strategies. It was hypothesized that astragalin might hinder the progression of AP by modulating the NLRP3 signaling pathway, and concurrently, gut microbiota was an indispensable part in the relief of pancreatic histopathological processes mediated by astragalin. Nevertheless, several limitations in our study deserve noting. Briefly, while murine models provide valuable mechanistic insights, they cannot fully recapitulate the complexity of human AP. Large-scale clinical studies are warranted to validate the translational applicability of these findings and substantiate the durability of the observed therapeutic effects. Also, the 12 key target genes identified through network pharmacology and transcriptomics were preliminarily validated by molecular docking. We acknowledge that functional experiments (e.g., gene silencing or overexpression of selected core targets) would offer deeper mechanistic insights. However, as the primary objective of this study was to elucidate the macro-regulatory role of astragalin on the gut microbiota and subsequent inflammatory responses, a detailed functional investigation of these downstream targets is beyond its current scope and constitutes a defined direction for our future research. Furthermore, while the optimal dose in this study was determined based on a comprehensive pharmacodynamic evaluation, future work should include systematic pharmacokinetic investigations to characterize the absorption, distribution, metabolism, and excretion of astragalin in AP model mice. Establishing a pharmacokinetic-pharmacodynamic (PK/PD) model will provide a stronger scientific basis for translational research from the preclinical to clinical stages. Additionally, while our multi-omics analyses used a rigorously controlled design with genetically identical subjects under standardized protocols and applied conservative statistical thresholds, the modest sample size (n = 3–5 per group) remains an important limitation that warrants validation through expanded studies. We emphasize that although the current findings are biologically plausible and demonstrate strong internal consistency, these results should be regarded as preliminary until replicated in more extensive investigations.

## Conclusion

This study innovatively combined network pharmacology, transcriptomics, metagenomics, metabolomics, and molecular biology research strategies to investigate the efficacy and mechanisms of astragalin in treating AP. The multifactorial mechanisms might be ascribed to the modulation of the pancreatic NLRP3 inflammatory signaling pathway and gut microbiota. These findings could provide clues for the future clinical practice of astragalin on AP. Furthermore, the research strategy utilized in this study could provide an exemplification for exploring the intestinal-pancreatic axis.

## Supplementary Information

Supplementary Material 1

Supplementary Material 2

Supplementary Material 3

Supplementary Material 4

## Data Availability

Data will be made available on request.

## Abbreviations

AP: Acute pancreatitis
TCM: Traditional Chinese medicine
AG: Astragalin
PAR2: Protease-Activated Receptor 2
iNOS: Inducible Nitric Oxide Synthase
MAPK: Mitogen-activated protein kinase
DEGs: Differentially expressed genes
FMT: Fecal microbiota transplantation
SPF: Specific-pathogen-free
H&E: Histological examination
IHC: Immunohistochemistry
DAPI: 4,6-Diamidino-2-phenylindole
MCC: Maximal Clique Centrality
TMM: Trimmed mean of M values
PLSDA: Partial Least Squares Discriminant Analysis
ANOSIM: Analysis of similarities
LEfSe: Linear discriminant analysis Effect Size
ANOVA: A one-way analysis of variance
NLRP signaling pathway: NOD-like receptor signaling pathway
SCFAs: Short chain fatty acids

## References

[CR1] Ba Q, Li M, Chen P, Huang C, Duan X, Lu L, Li J, Chu R, Xie D, Song H, Wu Y, Ying H, Jia X, Wang H (2017) Sex-dependent effects of cadmium exposure in early life on gut microbiota and fat accumulation in mice. Environ Health Perspect 125(3):437–446 27634282 10.1289/EHP360 PMC5332190

[CR2] Benjamini Y, Yekutieli D (2001) The control of the false discovery rate in multiple testing under dependency. Ann Stat 29(4):1165–1188

[CR3] Biczo G, Vegh ET, Shalbueva N, Mareninova OA, Elperin J, Lotshaw E, Gretler S, Lugea A, Malla SR, Dawson D, Ruchala P, Whitelegge J, French SW, Wen L, Husain SZ, Gorelick FS, Hegyi P, Rakonczay Z Jr, Gukovsky I, Gukovskaya AS (2018) Mitochondrial dysfunction, through impaired autophagy, leads to endoplasmic reticulum stress, deregulated lipid metabolism, and pancreatitis in animal models. Gastroenterology 154(3):689–703 29074451 10.1053/j.gastro.2017.10.012 PMC6369139

[CR4] Criddle DN, McLaughlin E, Murphy JA, Petersen OH, Sutton R (2007) The pancreas misled: signals to pancreatitis. Pancreatology 7(5–6):436–446. 10.1159/000108960 17898533 10.1159/000108960

[CR5] Deyaert S, Poppe J, Dai Vu L, Baudot A, Bubeck S, Bayne T, Krishnan K, Giusto M, Moltz S, Van den Abbeele P (2024) Functional muffins exert bifidogenic effects along with highly product-specific effects on the human gut microbiota ex vivo. Metabolites. 10.3390/metabo14090497 39330504 10.3390/metabo14090497 PMC11433953

[CR6] Etxeberria U, Fernandez-Quintela A, Milagro FI, Aguirre L, Martinez JA, Portillo MP (2013) Impact of polyphenols and polyphenol-rich dietary sources on gut microbiota composition. J Agric Food Chem 61(40):9517–9533 24033291 10.1021/jf402506c

[CR7] Fang F, Li Y, Lu X, Wu K, Zhou L, Sun Y, Wu J, Gao J (2023) Effect of potential postbiotics derived from food-isolated *Lactobacillus parabuchneri* on different enterotypes of human gut microbiome. Food Science and Technology/lebensmittel-Wissenschaft und-Technologie 182(000):8

[CR8] Frossard JL, Steer ML, Pastor CM (2008) Acute pancreatitis. Lancet 371(9607):143–152. 10.1016/S0140-6736(08)60107-5 18191686 10.1016/S0140-6736(08)60107-5

[CR9] Gao L, Dong X, Gong W, Huang W, Xue J, Zhu Q, Ma N, Chen W, Fu X, Gao X, Lin Z, Ding Y, Shi J, Tong Z, Liu T, Mukherjee R, Sutton R, Lu G, Li W (2021) Acinar cell NLRP3 inflammasome and gasdermin D (GSDMD) activation mediates pyroptosis and systemic inflammation in acute pancreatitis. Br J Pharmacol 178(17):3533–3552 33871879 10.1111/bph.15499

[CR10] Gukovskaya AS, Gukovsky I, Algul H, Habtezion A (2017) Autophagy, inflammation, and immune dysfunction in the pathogenesis of pancreatitis. Gastroenterology 153(5):1212–1226 28918190 10.1053/j.gastro.2017.08.071 PMC6338477

[CR11] Han HS, Hwang S, Choi SY, Hitayezu E, Humphrey MA, Enkhbayar A, Song DG, Kim M, Park JS, Park YT, Park JS, Cha KH, Choi KY (2024) *Roseburia intestinalis*-derived extracellular vesicles ameliorate colitis by modulating intestinal barrier, microbiome, and inflammatory responses. J Extracell Vesicles 13(8):e12487. 10.1002/jev2.12487 39166405 10.1002/jev2.12487 PMC11336657

[CR12] Jia L, Chen H, Yang J, Fang X, Niu W, Zhang M, Li J, Pan X, Ren Z, Sun J, Pan LL (2020) Combinatory antibiotic treatment protects against experimental acute pancreatitis by suppressing gut bacterial translocation to pancreas and inhibiting NLRP3 inflammasome pathway. Innate Immun 26(1):48–61 31615312 10.1177/1753425919881502 PMC6974879

[CR13] Jia Y, Shi Y, Wang J, Liu H, Huang Y, Wang H, Liu Y, Peng J (2024) Integrating metagenomics with metabolomics for gut microbiota and metabolites profiling in acute pancreatitis. Sci Rep 14(1):21491 39277616 10.1038/s41598-024-72057-z PMC11401878

[CR14] Kim D, Langmead B, Salzberg SL (2015) HISAT: a fast spliced aligner with low memory requirements. Nat Methods 12(4):357–360 25751142 10.1038/nmeth.3317 PMC4655817

[CR15] Lee MJ, Kang MJ, Lee SY, Lee E, Kim K, Won S, Suh DI, Kim KW, Sheen YH, Ahn K, Kim BS, Hong SJ (2018) Perturbations of gut microbiome genes in infants with atopic dermatitis according to feeding type. J Allergy Clin Immunol 141(4):1310–1319. 10.1016/j.jaci.2017.11.045 29339259 10.1016/j.jaci.2017.11.045

[CR16] Li XY, He C, Zhu Y, Lu NH (2020) Role of gut microbiota on intestinal barrier function in acute pancreatitis. World J Gastroenterol 26(18):2187–2193. 10.3748/wjg.v26.i18.2187 32476785 10.3748/wjg.v26.i18.2187 PMC7235204

[CR17] Li J, Shi B, Ren X, Hu J, Li Y, He S, Zhang G, Maolan A, Sun T, Qi X, Zhang X, Luo Y, Liu R, Hua B (2024) Lung-intestinal axis, Shuangshen granules attenuate lung metastasis by regulating the intestinal microbiota and related metabolites. Phytomedicine 132:155831 38908193 10.1016/j.phymed.2024.155831

[CR18] Liu Q, Ruan K, An Z, Li L, Ding C, Xu D, Yang J, Zhang X (2025) Updated review of research on the role of the gut microbiota and microbiota-derived metabolites in acute pancreatitis progression and inflammation-targeted therapy. Int J Biol Sci 21(3):1242–1258 39897025 10.7150/ijbs.108858 PMC11781165

[CR19] Louis P, Flint HJ (2017) Formation of propionate and butyrate by the human colonic microbiota. Environ Microbiol 19(1):29–41. 10.1111/1462-2920.13589 27928878 10.1111/1462-2920.13589

[CR20] Lugea A, Tischler D, Nguyen J, Gong J, Gukovsky I, French SW, Gorelick FS, Pandol SJ (2011) Adaptive unfolded protein response attenuates alcohol-induced pancreatic damage. Gastroenterology 140(3):987–997 21111739 10.1053/j.gastro.2010.11.038 PMC3057335

[CR21] Meng Y, Zhao M, Ma Q, Hua Q, Hu J, Zhou Q, Yi H, Zhang Z, Zhang L (2024) *Bifidobacterium bifidum* alleviates adenine-induced acute kidney injury in mice by improving intestinal barrier function. Food Funct 15(15):8030–8042 38984966 10.1039/d4fo02014f

[CR22] Mortazavi A, Williams BA, McCue K, Schaeffer L, Wold B (2008) Mapping and quantifying mammalian transcriptomes by RNA-Seq. Nat Methods 5(7):621–628 18516045 10.1038/nmeth.1226 PMC13303166

[CR23] Pan L, Yin N, Duan M, Mei Q, Zeng Y (2024) The role of gut microbiome and its metabolites in pancreatitis. mSystems 9(10):e0066524 39212377 10.1128/msystems.00665-24 PMC11494936

[CR24] Peng L, Gao X, Nie L, Xie J, Dai T, Shi C, Tao L, Wang Y, Tian Y, Sheng J (2020) Astragalin attenuates dextran sulfate sodium (DSS)-induced acute experimental colitis by alleviating gut microbiota dysbiosis and inhibiting NF-kappaB activation in mice. Front Immunol 11:2058 33042117 10.3389/fimmu.2020.02058 PMC7523281

[CR25] Pertea M, Pertea GM, Antonescu CM, Chang TC, Mendell JT, Salzberg SL (2015) StringTie enables improved reconstruction of a transcriptome from RNA-seq reads. Nat Biotechnol 33(3):290–295 25690850 10.1038/nbt.3122 PMC4643835

[CR26] Pertea M, Kim D, Pertea GM, Leek JT, Salzberg SL (2016) Transcript-level expression analysis of RNA-seq experiments with HISAT, StringTie and Ballgown. Nat Protoc 11(9):1650–1667. 10.1038/nprot.2016.095 27560171 10.1038/nprot.2016.095 PMC5032908

[CR27] Qi-Xiang M, Yang F, Ze-Hua H, Nuo-Ming Y, Rui-Long W, Bin-Qiang X, Jun-Jie F, Chun-Lan H, Yue Z (2022) Intestinal TLR4 deletion exacerbates acute pancreatitis through gut microbiota dysbiosis and Paneth cells deficiency. Gut Microbes. 10.1080/19490976.2022.2112882 35982604 10.1080/19490976.2022.2112882 PMC9397436

[CR28] Robinson MD, Oshlack A (2010) A scaling normalization method for differential expression analysis of RNA-seq data. Genome Biol 11(3):R25 20196867 10.1186/gb-2010-11-3-r25 PMC2864565

[CR29] Robinson MD, McCarthy DJ, Smyth GK (2010) edgeR: a Bioconductor package for differential expression analysis of digital gene expression data. Bioinformatics 26(1):139–140 19910308 10.1093/bioinformatics/btp616 PMC2796818

[CR30] Schmidt J, Rattner DW, Lewandrowski K, Compton CC, Mandavilli U, Knoefel WT, Warshaw AL (1992) A better model of acute pancreatitis for evaluating therapy. Ann Surg 215(1):44–56 1731649 10.1097/00000658-199201000-00007 PMC1242369

[CR31] Sendler M, van den Brandt C, Glaubitz J, Wilden A, Golchert J, Weiss FU, Homuth G, De Freitas Chama LL, Mishra N, Mahajan UM, Bossaller L, Volker U, Broker BM, Mayerle J, Lerch MM (2020) NLRP3 inflammasome regulates development of systemic inflammatory response and compensatory anti-inflammatory response syndromes in mice with acute pancreatitis. Gastroenterology 158(1):253-269 e14 31593700 10.1053/j.gastro.2019.09.040

[CR32] Shen Z, Luo W, Tan B, Nie K, Deng M, Wu S, Xiao M, Wu X, Meng X, Tong T, Zhang C, Ma K, Liao Y, Xu J, Wang X (2022) *Roseburia intestinalis* stimulates TLR5-dependent intestinal immunity against Crohn’s disease. EBioMedicine 85:104285 36182776 10.1016/j.ebiom.2022.104285 PMC9526137

[CR33] Shkoporov AN, Chaplin AV, Khokhlova EV, Shcherbakova VA, Motuzova OV, Bozhenko VK, Kafarskaia LI, Efimov BA (2015) Alistipes inops sp. nov. and Coprobacter secundus sp. nov. isolated from human faeces. Int J Syst Evolutionary Microbiol 65(12):4580–4588 10.1099/ijsem.0.000617 26377180

[CR34] Song TJ, Ke J, Chen F, Zhang JY, Zhang C, Chen HY (2022) Effect of SNHG11/miR-7-5p/PLCB1 axis on acute pancreatitis through inhibiting p38MAPK pathway. Cells. 10.3390/cells12010065 36611865 10.3390/cells12010065 PMC9818913

[CR35] Soromou LW, Chen N, Jiang L, Huo M, Wei M, Chu X, Millimouno FM, Feng H, Sidime Y, Deng X (2012) Astragalin attenuates lipopolysaccharide-induced inflammatory responses by down-regulating NF-kappaB signaling pathway. Biochem Biophys Res Commun 419(2):256–261 22342978 10.1016/j.bbrc.2012.02.005

[CR36] Steele CW, Karim SA, Foth M, Rishi L, Leach JD, Porter RJ, Nixon C, Jeffry Evans TR, Carter CR, Nibbs RJ, Sansom OJ, Morton JP (2015) CXCR2 inhibition suppresses acute and chronic pancreatic inflammation. J Pathol 237(1):85–97 25950520 10.1002/path.4555 PMC4833178

[CR37] Tang E, Lin H, Yang Y, Xu J, Lin B, Yang Y, Huang Z, Wu X (2024) Dietary astragalin confers protection against lipopolysaccharide-induced intestinal mucosal barrier damage through mitigating inflammation and modulating intestinal microbiota. Front Nutr 11:1481203 39421621 10.3389/fnut.2024.1481203 PMC11483603

[CR38] Tintore M, Cune J, Vu LD, Poppe J, Van den Abbeele P, Baudot A, de Lecea C (2024) A long-chain dextran produced by *Weissella cibaria* boosts the diversity of health-related gut microbes ex vivo. Biology. 10.3390/biology13010051 38248481 10.3390/biology13010051 PMC10813514

[CR39] Tong Y, Fu H, Xia C, Song W, Li Y, Zhao J, Zhang X, Gao X, Yong J, Liu Q, Yang C, Wang H (2020) Astragalin exerted antidepressant-like action through SIRT1 signaling modulated NLRP3 inflammasome deactivation. ACS Chem Neurosci 11(10):1495–1503 32364698 10.1021/acschemneuro.0c00156

[CR40] Wang L, Li Y, Ma Q, Liu Y, Rui YY, Xue P, Zhou ZG (2011) Chaiqin chengqi decoction decreases IL-6 levels in patients with acute pancreatitis. J Zhejiang Univ Sci B 12(12):1034–1040 22135153 10.1631/jzus.B1000406 PMC3232437

[CR41] Wang D, Wang L, Han L, Wang B, Shi R, Ye J, Xia B, Zhao Z, Zhao B, Liu X (2023) Leucine-restricted diet ameliorates obesity-linked cognitive deficits: involvement of the microbiota–gut–brain axis. J Agric Food Chem 71(24):9404–9418 37306277 10.1021/acs.jafc.3c01524

[CR42] Wang LJ, Jin YL, Pei WL, Li JC, Zhang RL, Wang JJ, Lin W (2024) Amuc_1100 pretreatment alleviates acute pancreatitis in a mouse model through regulating gut microbiota and inhibiting inflammatory infiltration. Acta Pharmacol Sin 45(3):570–580 38012292 10.1038/s41401-023-01186-4 PMC10834448

[CR43] Wu X, Yao J, Hu Q, Kang H, Miao Y, Zhu L, Li C, Zhao X, Li J, Wan M, Tang W (2022) Emodin ameliorates acute pancreatitis-associated lung injury through inhibiting the alveolar macrophages pyroptosis. Front Pharmacol 13:873053. 10.3389/fphar.2022.873053 35721108 10.3389/fphar.2022.873053 PMC9201345

[CR44] Xia Q, Deng LH (2013) [Hot issues on the treatment of severe acute pancreatitis by Integrated Traditional Chinese and Western Medicine]. Sichuan Da Xue Xue Bao Yi Xue Ban 44(6):962–965 24490512

[CR45] Xing F, Geng L, Guan H, Liu D, Li Y, Zeng L, Chen Y, Tian R, Li Z, Cao R, Zhao Y, Yan P, Qiang H, Kong N, Wang K, Yang P (2022) Astragalin mitigates inflammatory osteolysis by negatively modulating osteoclastogenesis via ROS and MAPK signaling pathway. Int Immunopharmacol 112:109278 36215870 10.1016/j.intimp.2022.109278

[CR46] Yan X, Li J, Wu D (2023) The role of short-chain fatty acids in acute pancreatitis. Molecules. 10.3390/molecules28134985 37446647 10.3390/molecules28134985 PMC10343743

[CR47] Yang X, Kui L, Tang M, Li D, Wei K, Chen W, Miao J, Dong Y (2020) High-throughput transcriptome profiling in drug and biomarker discovery. Front Genet 11:19. 10.3389/fgene.2020.00019 32117438 10.3389/fgene.2020.00019 PMC7013098

[CR48] Yuan H, Ma Q, Cui H, Liu G, Zhao X, Li W, Piao G (2017) How can synergism of traditional medicines benefit from network pharmacology? Molecules. 10.3390/molecules22071135 28686181 10.3390/molecules22071135 PMC6152294

[CR49] Zagato E, Pozzi C, Bertocchi A, Schioppa T, Saccheri F, Guglietta S, Fosso B, Melocchi L, Nizzoli G, Troisi J, Marzano M, Oresta B, Spadoni I, Atarashi K, Carloni S, Arioli S, Fornasa G, Asnicar F, Segata N, Guglielmetti S, Honda K, Pesole G, Vermi W, Penna G, Rescigno M (2020) Endogenous murine microbiota member *Faecalibaculum rodentium* and its human homologue protect from intestinal tumour growth. Nat Microbiol 5(3):511–524 31988379 10.1038/s41564-019-0649-5 PMC7048616

[CR50] Zhang W, Lu X, Wang W, Ding Z, Fu Y, Zhou X, Zhang N, Cao Y (2017) Inhibitory effects of emodin, thymol, and astragalin on *Leptospira interrogans*-induced inflammatory response in the uterine and endometrium epithelial cells of mice. Inflammation 40(2):666–675 28210912 10.1007/s10753-017-0513-9

[CR51] Zhang XM, Zhang ZY, Zhang CH, Wu J, Wang YX, Zhang GX (2018) Intestinal microbial community differs between acute pancreatitis patients and healthy volunteers. Biomed Environ Sci 31(1):81–86. 10.3967/bes2018.010 29409589 10.3967/bes2018.010

[CR52] Zhang C, Li G, Lu T, Liu L, Sui Y, Bai R, Li L, Sun B (2023) The interaction of microbiome and pancreas in acute pancreatitis. Biomolecules. 10.3390/biom14010059 38254659 10.3390/biom14010059 PMC10813032

[CR53] Zhao LJ, Chen P, Huang L, He WQ, Tang YR, Wang R, Luo ZL, Ren JD (2024) Heparan sulfate acts as an activator of the NLRP3 inflammasome promoting inflammatory response in the development of acute pancreatitis. J Gastroenterol 59(9):869–879 38864913 10.1007/s00535-024-02127-6

[CR54] Zhou Q, Tao X, Guo F, Wu Y, Deng D, Lv L, Dong D, Shang D, Xiang H (2023) Tryptophan metabolite norharman secreted by cultivated *Lactobacillus* attenuates acute pancreatitis as an antagonist of histone deacetylases. BMC Med 21(1):329 37635214 10.1186/s12916-023-02997-2 PMC10463520

[CR55] Zhu H, Wang S, Shan C, Li X, Tan B, Chen Q, Yang Y, Yu H, Yang A (2021) Mechanism of protective effect of xuan-bai-cheng-qi decoction on LPS-induced acute lung injury based on an integrated network pharmacology and RNA-sequencing approach. Respir Res 22(1):188 34183011 10.1186/s12931-021-01781-1 PMC8237774

