# Supplementary material for: Astragalin attenuates caerulein-induced acute pancreatitis by targeting the NLRP3 signaling pathway and gut microbiota
Source: Bioresour Bioprocess. 2025 Dec 3;12(1):139. doi: 10.1186/s40643-025-00977-3 (PMC12675899; doi:10.1186/s40643-025-00977-3)
Supplement: Supplementary file 1 — Supplementary Material 1 [file 40643_2025_977_MOESM1_ESM.docx]

**Supplementary figures**


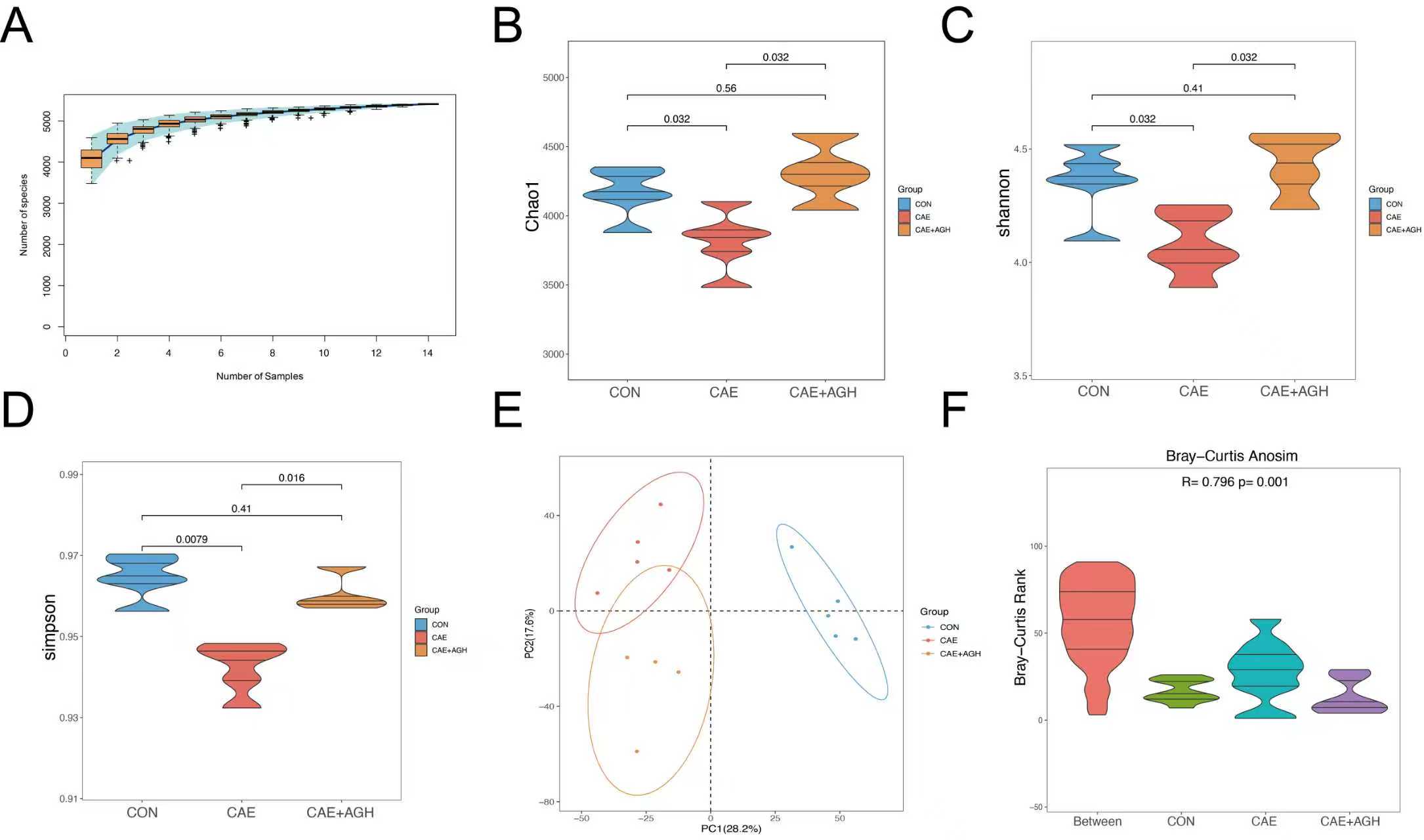


Fig. S1 Effects of astragalin on the microbial diversity in AP mice.

(A) Species accumulation curve. (B-D) Alpha diversity indexes. (E) PLSDA analysis. (F) ANOSIM analysis. N=4-5.


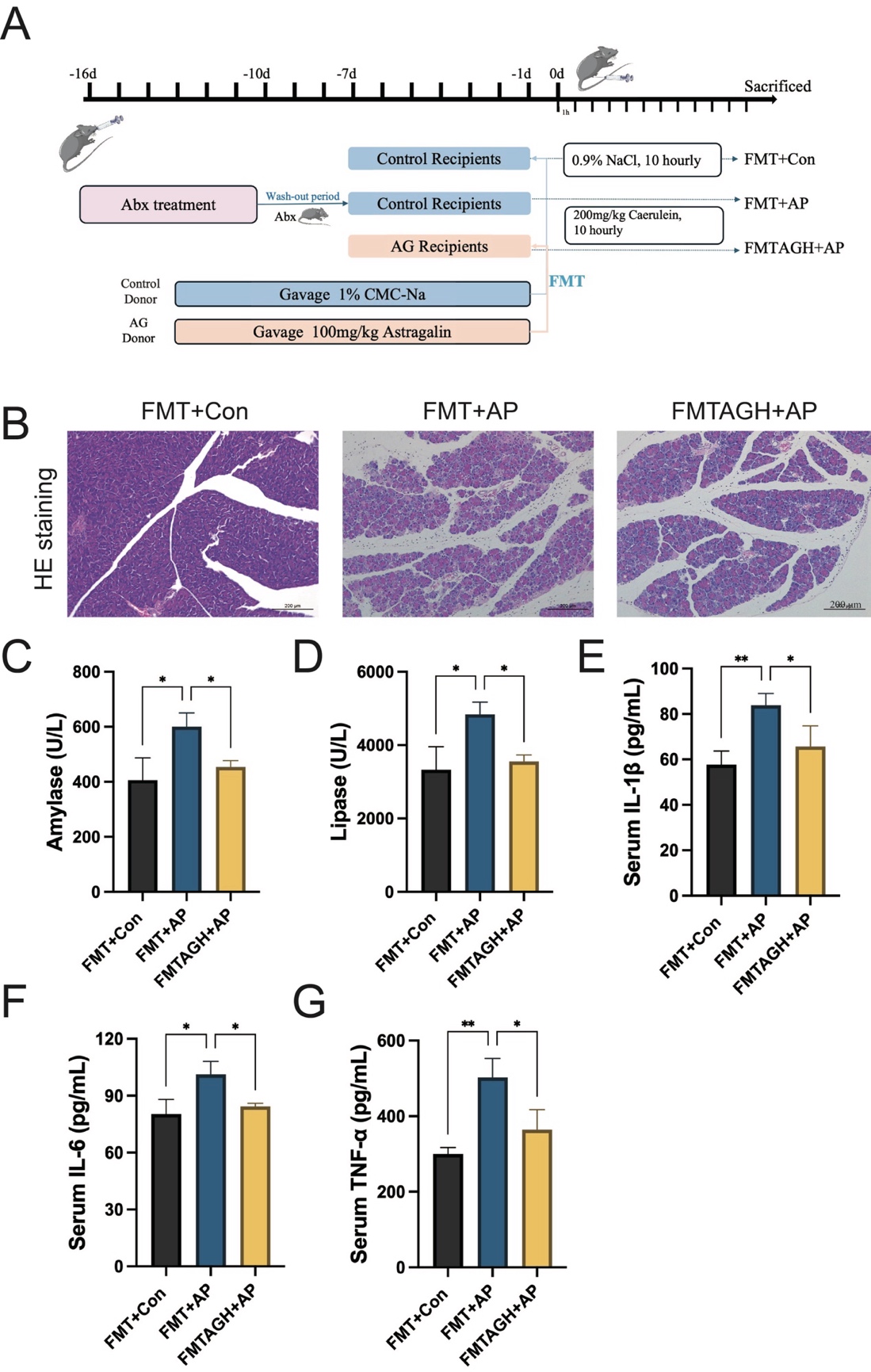


Fig. S2 Effects of astragalin in AP mice are transferable by FMT.

(A) Schematic diagram of fecal microbiota transplantation. (B) H&E staining. (C and D) Serum amylase and lipase. (E-G) Serum inflammatory cytokines. Data are expressed as mean ± SD of independent experiments. N=5. **P* < 0.05, ***P* < 0.01 (versus the FMT+AP group).


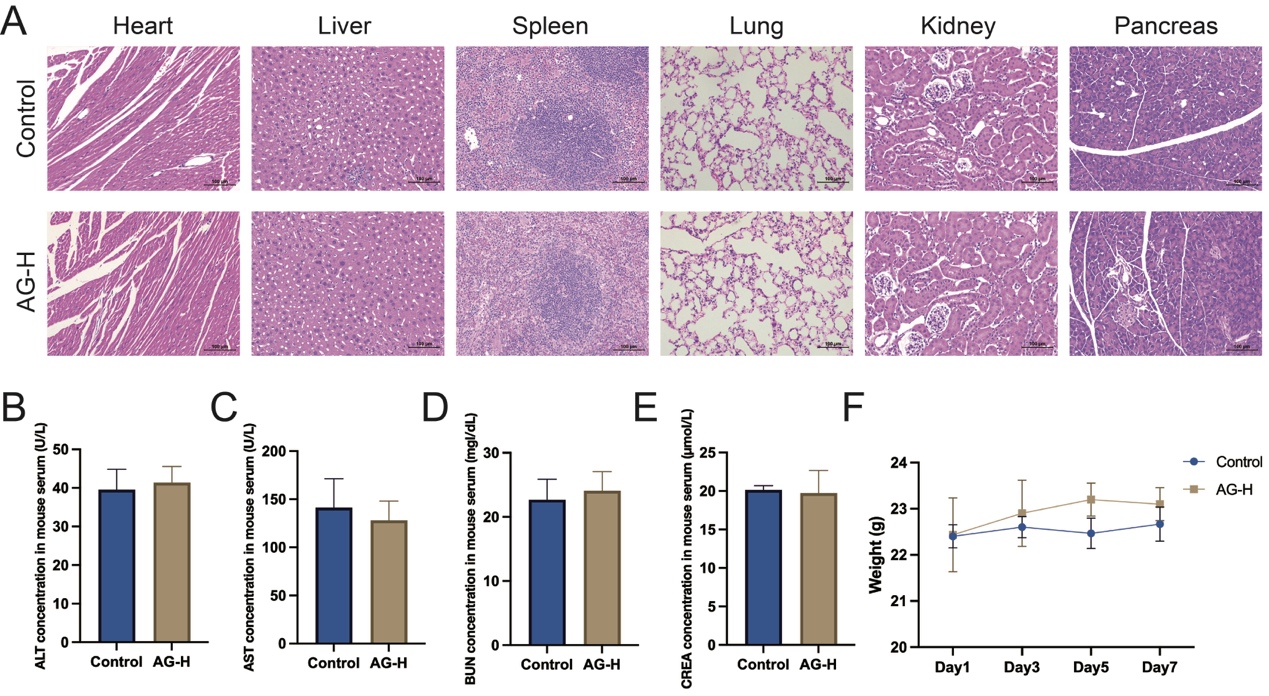


Fig. S3 Astragalin gavage showed no significant toxicity in vivo.

1. H&E staining of the heart, liver, spleen, lung, kidney, and pancreas. (B-E) Indicators of hepatic and renal function. (F) Body weight. Data are expressed as mean ± SD of independent experiments. N=5.
