# Supplementary figures and images for: Astragalin attenuates caerulein-induced acute pancreatitis by targeting the NLRP3 signaling pathway and gut microbiota
Source: Bioresour Bioprocess. 2025 Dec 3;12(1):139. doi: 10.1186/s40643-025-00977-3 (PMC12675899; doi:10.1186/s40643-025-00977-3)

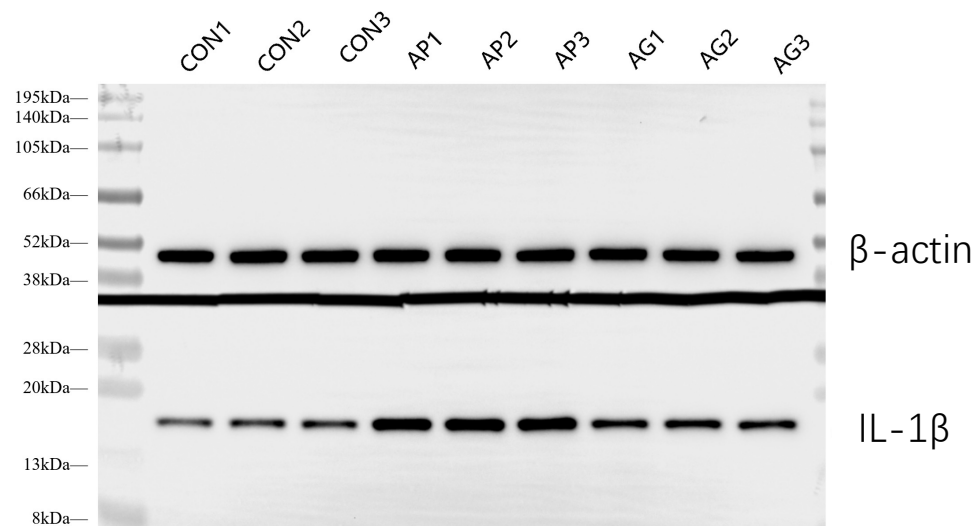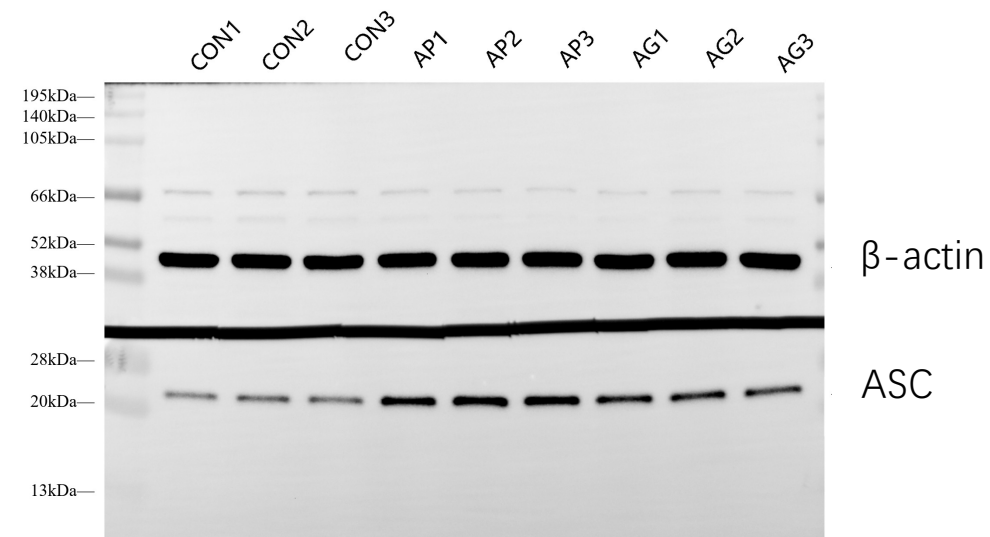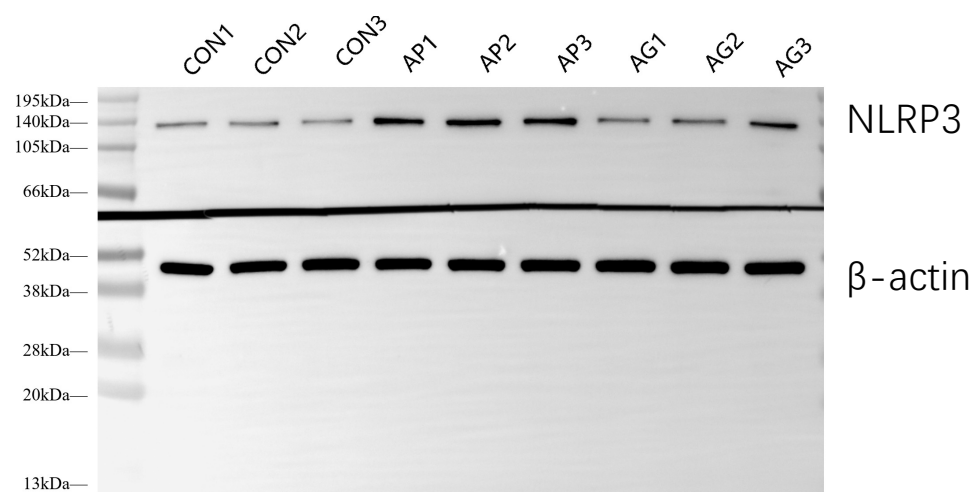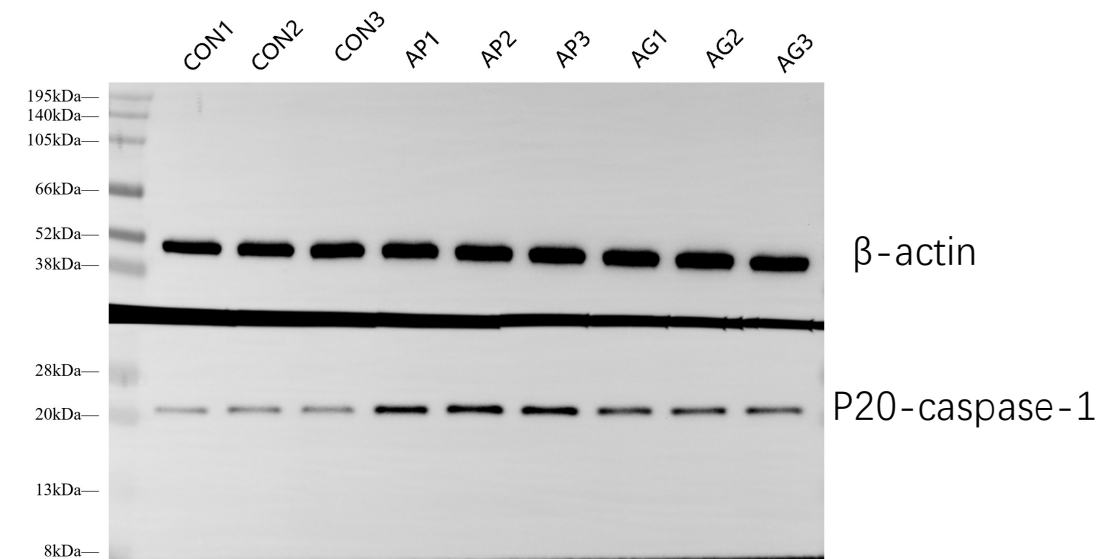

Supplement: Supplementary file 4 — Supplementary Material 4 [file 40643_2025_977_MOESM4_ESM.pdf]
